# Efficient deletion of microRNAs using CRISPR/Cas9 with dual guide RNAs

**DOI:** 10.3389/fmolb.2023.1295507

**Published:** 2024-04-02

**Authors:** Smitha Ijee, Karthik Chambayil, Anurag Dutta Chaudhury, Abhirup Bagchi, Kirti Modak, Saswati Das, Esther Sathya Bama Benjamin, Sonam Rani, Daniel Zechariah Paul, Aneesha Nath, Debanjan Roy, Dhavapriya Palani, Sweety Priyanka, Rakshini Ravichandran, Betty K. Kumary, Yazhini Sivamani, Vijayanand S., Dinesh Babu, Yukio Nakamura, Vasanth Thamodaran, Poonkuzhali Balasubramanian, Shaji R. Velayudhan

**Affiliations:** ^1^ Centre for Stem Cell Research (A Unit of inStem, Bengaluru), Christian Medical College Campus, Vellore, India; ^2^ Department of Biotechnology, Thiruvalluvar University, Vellore, India; ^3^ Sree Chitra Tirunal Institute of Science and Medical Technology, Thiruvananthapuram, India; ^4^ Department of Haematology, Christian Medical College Campus, Vellore, India; ^5^ Regional Centre for Biotechnology, New Delhi, India; ^6^ Manipal Academy of Higher Education, Manipal, India; ^7^ Cell Engineering Division, RIKEN BioResource Research Center, Tsukuba, Japan; ^8^ Tata Institute of Genetics and Society, Bengaluru, India

**Keywords:** miRNA, CRISPR-Cas9, dual-gRNA, iPSCs, erythroid

## Abstract

MicroRNAs (miRNAs) are short non-coding RNAs that play crucial roles in gene regulation, exerting post-transcriptional silencing, thereby influencing cellular function, development, and disease. Traditional loss-of-function methods for studying miRNA functions, such as miRNA inhibitors and sponges, present limitations in terms of specificity, transient effects, and off-target effects. Similarly, CRISPR/Cas9-based editing of miRNAs using single guide RNAs (sgRNAs) also has limitations in terms of design space for generating effective gRNAs. In this study, we introduce a novel approach that utilizes CRISPR/Cas9 with dual guide RNAs (dgRNAs) for the rapid and efficient generation of short deletions within miRNA genomic regions. Through the expression of dgRNAs through single-copy lentiviral integration, this approach achieves over a 90% downregulation of targeted miRNAs within a week. We conducted a comprehensive analysis of various parameters influencing efficient deletion formation. In addition, we employed doxycycline (Dox)-inducible expression of Cas9 from the AAVS1 locus, enabling homogeneous, temporal, and stage-specific editing during cellular differentiation. Compared to miRNA inhibitory methods, the dgRNA-based approach offers higher specificity, allowing for the deletion of individual miRNAs with similar seed sequences, without affecting other miRNAs. Due to the increased design space, the dgRNA-based approach provides greater flexibility in gRNA design compared to the sgRNA-based approach. We successfully applied this approach in two human cell lines, demonstrating its applicability for studying the mechanisms of human erythropoiesis and pluripotent stem cell (iPSC) biology and differentiation. Efficient deletion of miR-451 and miR-144 resulted in blockage of erythroid differentiation, and the deletion of miR-23a and miR-27a significantly affected iPSC survival. We have validated the highly efficient deletion of genomic regions by editing protein-coding genes, resulting in a significant impact on protein expression. This protocol has the potential to be extended to delete multiple miRNAs within miRNA clusters, allowing for future investigations into the cooperative effects of the cluster members on cellular functions. The protocol utilizing dgRNAs for miRNA deletion can be employed to generate efficient pooled libraries for high-throughput comprehensive analysis of miRNAs involved in different biological processes.

## Introduction

It has been estimated that approximately 20,000 protein-coding genes constitute only 2% of the human genome, with the remainder being those encoding non-coding RNAs (Carninci et al., 2005). MicroRNAs (miRNAs) are a class of short non-coding RNAs (20–24 nt) that regulate gene expression at the post-transcriptional and translational levels by binding to the 3′ untranslated regions of messenger RNAs (mRNAs) (Bassett et al., 2014). They play crucial roles in cellular function, development, and disease (Gebert and MacRae, 2019). Due to the short seed sequences that determine miRNA target recognition, a single miRNA can regulate hundreds to thousands of target mRNAs, while multiple miRNAs can regulate a single mRNA (Kehl et al., 2017). It is estimated that miRNAs can regulate up to 60% of all human protein-coding genes (Friedman et al., 2009).

Despite identifying over 3,500 human miRNAs across various cell types (Kozomara and Griffiths-Jones, 2014), the biological functions of the majority of miRNAs remain unknown. Functional characterization of miRNAs can be accomplished using gain-of-function and loss-of-function approaches. Gain-of-function methods involve the introduction of miRNAs into cells either through the transfection of double-stranded miRNA mimics (Nogimori et al., 2019) or through the utilization of vectors for miRNA expression (Fan et al., 2019). Loss-of-function approaches rely on synthetic antisense oligonucleotide miRNA inhibitors, which bind to and inhibit endogenous miRNAs, preventing their interaction with the target mRNAs (Stenvang et al., 2012; Lima et al., 2018; Bajan and Hutvagner, 2020). Additionally, miRNA sponges and decoys, which contain multiple binding sites of miRNAs, competitively inhibit endogenous miRNAs by sequestering them, thereby reducing their availability to bind target mRNAs and dampening their regulatory activity (Ebert et al., 2007; Gentner et al., 2009). Nonetheless, these methodologies have certain limitations, such as transient effects, specificity concerns, toxicity, and variable efficiency (Sayed et al., 2021; Bayraktar et al., 2023). Additionally, given the presence of highly homologous seed sequences among some miRNAs, ensuring specific repression of the targeted miRNAs is crucial to avoid biased phenotypic outcomes (Stenvang et al., 2012).

The CRISPR/Cas9 genome editing tool, which employs Cas9 nuclease and guide RNAs (gRNAs) to modify the specific genomic regions, operates by inducing DNA double-strand breaks (DSBs) and relies on error-prone non-homologous end joining (NHEJ) DNA repair, leading to the efficient generation of small insertions and deletions (indels) at the targeted sites (Ran et al., 2013). Recent studies have utilized CRISPR/Cas9 with single guide RNAs (sgRNAs) targeting crucial functional sequences of miRNAs, including their seed sequences, loop regions, or biogenesis processing sites. The results demonstrated significant reductions in the expression of the targeted miRNAs (Jiang et al., 2014; Chang et al., 2016; Wallace et al., 2016; Huo et al., 2017; Zhang et al., 2018; Li et al., 2020). Importantly, unlike miRNA inhibitory methods that employ antisense oligonucleotides to deplete miRNA transcripts, CRISPR/Cas9 genomic editing leads to permanent disruption of miRNA expression. However, genome editing might inadvertently yield unpredictable and unintended indels, potentially outside critical regions for miRNA function, thus not disrupting the expression of targeted miRNAs (Bi et al., 2020; Vidigal and Ventura, 2014; Ferreira and Reis, 2023). Furthermore, indels occurring within the stem regions of miRNAs can give rise to non-naturally occurring miRNA transcripts (Bhattacharya et al., 2012). Although CRISPR/Cas9 mediated mutations at miRNA biogenesis processing sites have demonstrated the capability to reduce mature miRNAs by as much as 90% (Chang et al., 2016), a significant challenge arises in designing suitable gRNAs for all the miRNAs under study due to the limited design space available for this approach. We were unable to identify suitable gRNAs at the biogenesis sites for more than 40% of the miRNAs that we analyzed.

In this study, we report the development of a rapid and efficient protocol utilizing dual gRNAs (dgRNAs) to generate short deletions in human miRNA genomic regions. When two gRNAs bind to a genomic region, a short distance apart, they can trigger the simultaneous creation of DSBs, resulting in the generation of deletions between the binding sites of these gRNAs (Aparicio-prat et al., 2016). This method has been used to generate deletions within long non-coding RNAs (Yin et al., 2015; Aparicio-prat et al., 2016; Zhu et al., 2016; Chaudhary et al., 2017; Hao et al., 2020). Zhu et al. demonstrated efficient deletion formation (>90%) within a pool of cells that were lentivirally transduced with dgRNAs targeting one specific non-coding RNA (Zhu et al., 2016). However, this high efficiency required more than 2 weeks to achieve, and it remains uncertain whether comparable levels of efficiency could be attained for multiple non-coding RNAs. Deletion of miRNAs in mammalian cells using dgRNA expression vectors showed extremely low efficiency, necessitating single-cell sorting to isolate clones with the deletions of the targeted miRNAs for subsequent functional studies (Ho et al., 2015; Liu et al., 2016; Yan et al., 2019; Hong et al., 2020). Therefore, we assessed various parameters, including the on-target efficiency of gRNAs and the orientation and distance between the dgRNAs, which influence the efficiency of deletion formation. Through single-copy lentiviral integration of dgRNAs, we achieved a downregulation of over 90% for the targeted miRNAs within a week. The utilization of the CRISPR/Cas9 dgRNA strategy enables the specific targeting and investigation of individual miRNAs. This approach holds significant promise for gaining valuable insights into the functional roles of these molecules.

## Methods

### Cell culture

HUDEP2 (HUDEP-2) cells (a kind gift from Yukio Nakamura) (Kurita et al., 2013) were cultured following previously reported protocols (Kurita et al., 2013; Bagchi et al., 2021). Briefly, the cells were expanded in StemSpan SFEM-II medium (Stem Cell Technologies) supplemented with 1 µM dexamethasone, 1 μg/mL doxycycline (Dox), 50 ng/mL recombinant human stem cell factor (rh SCF), 3 units/mL recombinant human erythropoietin (rh EPO), 10 ng/mL recombinant human interleukin 3 (rh IL-3) and 1% penicillin/streptomycin. The AAVS1-Tet-On-Cas9 iPSCs generated in our laboratory (Thamodaran et al., 2022) were cultured in Matrigel (Corning) - coated plates containing mTeSR medium (STEMCELL Technologies). Upon reaching 70%–80% confluency, colonies were dissociated with Versene (Thermo Fisher Scientific) following the manufacturer’s protocol and passaged at a 1:4 ratio.

### Differentiation of HUDEP-2 cells

The differentiation of HUDEP-2 cells was performed following a previously described protocol (Hawksworth et al., 2018; Bagchi et al., 2021). Initially, the cells were seeded at a density of 2 × 10^5^ cells/mL in the differentiation medium consisting of Iscove’s Modified Dulbecco’s Medium (IMDM) supplemented with 2% (v/v) fetal bovine serum (Thermo Fischer Scientific), 3% (v/v) AB Serum (MP Biomedicals), 10 μg/mL insulin (Sigma Aldrich), 3 U/mL heparin (Sigma Aldrich), 200 μg/mL human holotransferrin (Sigma Aldrich), 1 ng/mL rh IL-3 (Peprotech), 10 ng/mL rh SCF (Peprotech), 3 U/mL rh Epo (Peprotech) and 1 μg/mL Dox (Sigma-Aldrich). After 2 days, the cells were reseeded at a density of 3.5 × 10^5^ cells/mL in a fresh medium. On day 4, the cells were further reseeded at a density of 5 × 10^5^ cells/mL in a fresh medium without Dox. Subsequently, on day 6, a complete medium change was performed, and the cells were reseeded at a density of 1 × 10^6^ cells/mL with an increased concentration of holotransferrin (500 μg/mL). On day 8, the cells were transferred to a differentiation medium devoid of rh SCF, rh IL-3, or Dox and maintained at a density of 1 × 10^6^ cells/mL with complete medium changes every 2 days until day 10.

### gRNA design

The stem-loop and flanking 30 nucleotide sequences of miRNAs were obtained from miRBase v21.1 (Kozomara and Griffiths-Jones, 2014) and the UCSC genome browser. To design the gRNAs, we utilized CRISPOR (Concordet and Haeussler, 2018) (http://crispor.tefor.net/), which provided on-target editing efficiency (Doench et al., 2016) and off-target effect scores. For miRNA knockout experiments using sgRNAs, we designed the gRNAs to be adjacent to the miRNA seed sequences or Drosha and Dicer biogenesis processing sites. For dgRNA knockout experiments, two gRNAs were designed to encompass the genomic sequences of either the 5p or 3p arms of the miRNAs. The gRNAs designed to be cloned in pKLV2.2 lentiviral vectors were synthesized commercially. The complimentary oligos, to be cloned into the hU6gRNA5(*Bbs*I) cassette of the pKLV2.2 vectors were synthesized with tag sequences top strand oligo- 5′- CACCNNNNNNNNNNNNNNNNNNNN-3′ and bottom strand oligo—3′-NNNNNNNNNNNNNNNNNNNNCAAA-5′. The complimentary oligos, to be cloned into the h7SKgRNA5(*Bbs*I) cassette were synthesized with tag sequences in the top strand oligo and bottom strand oligo as described earlier (Tzelepis et al., 2016): Top strand oligo- 5′- CTCNNNNNNNNNNNNNNNNNNNN-3′ and bottom strand oligo—3′-NNNNNNNNNNNNNNNNNNNNCAA-5′. The overhangs for cloning in LentiCRISPR V2 and pL.CRISPR.EFS.GFP lentiviral vectors were designed as described earlier (Tzelepis et al., 2016; Heckl et al., 2014). 

### Generation of single gRNA lentiviral vectors

Single gRNAs targeting the miRNA regions were cloned into LentiCRISPR V2 (a gift from Feng Zhang, Addgene plasmid #52961) or pKLV2.2-h7SKgRNA5(SapI)-hU6gRNA5(BbsI)-PGKpuroBFP-W (Addgene plasmid # 72666) or pKLV2.2-mU6gRNA5(SapI)-hU6gRNA5(BbsI)-PGKpuroBFP-W (Addgene no#72666) (gifts from Kosuke Yusa) (Tzelepis et al., 2016; Ihry et al., 2018). The gRNAs were cloned at the *BsmB*I site for LentiCRISPR V2 plasmid and *Sap*I or *Bbs*I sites for the pKLV2.2 plasmids as previously described (Sanjana et al., 2014; Tzelepis et al., 2016). Single gRNAs targeting the γ globin promoter region were cloned into pL.CRISPR.EFS.GFP (Addgene plasmid #57818) (gift from Benjamin Ebert) at the *Bsm*BI site (Heckl et al., 2014).

### Generation of dgRNA expression lentiviral vectors

To generate pKLV2.2 dgRNA expression lentiviral vectors, the dgRNAs were cloned in the pKLV2.2-h7SKgRNA5(SapI)-hU6gRNA5(BbsI)-PGKpuroBFP-W (Addgene plasmid # 72666; a gift from Kosuke Yusa) (Tzelepis et al., 2016). The plasmid was digested with *Sap*I first for cloning the first gRNA and then digested with *Bbs*I for cloning the second gRNA. The oligos for gRNA1 and gRNA2 were synthesized with specific 5′ overhangs for cloning at the *Sap*I and *Bbs*I sites of the plasmid (Tzelepis et al., 2016).

### Preparation of lentiviruses

For the generation of lentiviruses, the gRNA and Cas9 expression plasmids were co-transfected with pMD2.G envelope plasmid (Addgene 12259) and psPAX2 packaging plasmid (Addgene 12260) (gifts from Didier Trono). The virus supernatants were collected after 48, 60 and 72 h, pooled together, and concentrated 100 times using the Lenti-X Concentrator (Takara Bio), and stored at −80°C as aliquots. Approximately 1–2 × 10^5^ HUDEP2 cells/mL were transduced with the lentiviruses in the presence of 8 μg/mL polybrene (Sigma-Aldrich) by spinfection at 2,250 rpm for 1.5 h at room temperature.

### Generation of Cas9 expressing cell lines

Cas9-HUDEP2 cells were generated by transducing HUDEP2 cells with pLentiCas9-T2A-BFP lentiviral vector (Addgene 78547, a gift from Roderic Guigo and Rory Johnson). The transduced cells that express blue fluorescence protein (BFP+ cells) were flow-sorted and further selected with 10 μg/mL blasticidin to ensure uniform Cas9 expression in the cells. Cas9-THP1, Cas9-K562, and Cas9-EM2 cell lines were generated by transducing THP1, K562, and EM2 cells, respectively, with pCLIP-Cas9-Nuclease-EFS-Blasticidin (Transomic, Cat No. V085) lentiviral vector, and the transduced cells were selected with 2 μg/mL of blasticidin from day 5 after transduction for 10 days. AAVS1-TetOn-Cas9-HUDEP2 cells were generated by transfecting HUDEP2 cells with 3 plasmids, pAAVS1-PDi-CRISPRn (Addgene ID 73500, gift from Bruce Conklin Bruce), pZT-AAVS1-R1 (Addgene ID 52638, gift from Mahendra Rao and Jizhong Zou) and pZT-AAVS1-L1 (Addgene ID 52637, gift from Mahendra Rao and Jizhong Zou) as described earlier (Luo et al., 2014; Thamodaran et al., 2022). The cells with the successful integration of the Tet-On-Cas9 cassette at the AAVS1 site were selected by puromycin selection. The puromycin-selected cells were further subjected to single-cell sorting, and the single-cell clones were propagated in the presence of puromycin to develop clonal cell lines. One of the clones exhibiting homogeneous Cas9 expression referred to as AAVS1-TetOn-Cas9-HUDEP2 cells, was selected for genome editing experiments.

### Transduction of gRNA lentiviral vectors in cell lines

Concentrated viruses generated with pLentiCRISPRV2 and pKLV2.2 lentiviral vectors with cloned sgRNAs were transduced into 2 × 10^5^ HUDEP2 and Cas9-HUDEP2 cells/mL, respectively, in the presence of 8 μg/mL polybrene by spinfection at 2,250 rpm for 1.5 h at room temperature. After 5 days, puromycin selection (for pLentiCRISPRV2) or flow sorting of BFP+ cells (for pKLV2.2) was performed to select the transduced cells. Cas9-HUDEP2 cells were transduced with LVMUsg2PG dual-gRNA lentiviral vectors and the cells that expressed green fluorescence protein (GFP+ cells) were flow-sorted and cultured for 3–4 weeks to estimate the mutations in the targeted regions. Similarly, AAVS1-Tet-On-Cas9-HUDEP2 cells were transduced with pKLV2.2 dgRNA viruses, and the BFP^+^ transduced cells were flow-sorted 5–6 days after transduction to determine the formation of deletions in the targeted regions. AAVS1-Tet-On-Cas9 iPSCs (Thamodaran et al., 2022) were cultured in mTeESR medium (Stem Cell Technologies) in 12 well plates. When the cells reached ∼75% confluency, they were transduced with pKLV2.2 dgRNA lentiviruses. After 5–6 days of transduction, the BFP+ transduced cells were flow-sorted and cultured in the presence and absence of Dox. DNA samples were collected on day 3 and day 6 after Dox supplementation for mutation analysis. Similarly, the Cas9-THP1, Cas9-K562, and Cas9-EM2 cell lines were transduced with pKLV2.2 dgRNA viruses in the presence of 8 μg/mL polybrene by spinfection at 2,250 rpm for 1.5 h at room temperature. After 6 days, BFP^+^ cells were flow sorted and further cultured to estimate mutations caused by the dgRNAs at the targeted genomic region.

### CRISPR/Cas9 mutation analysis by T7EN1 assay and DECODR

Genomic DNA was extracted from the transduced cells using Gentra Puregene High Molecular Weight DNA Extraction Kit (Qiagen). DNA fragments spanning the target sites of CRISPR/cas9 were amplified using EmeraldAmp GT PCR Master Mix (Takara Bio), and PCR products were purified using a PCR product purification Kit (Machery Nagel). For the T7EN1 assay, 200–500 ng of purified PCR products in NEBuffer 2 (New England Biolabs) were denatured at 95°C for 5 min, then annealed at 95°C–85°C at 2°C/s, followed by 85°C–25°C at −0.1°C/s in a thermocycler (Y. Niu et al., 2014). Subsequently, 10 units of T7EN1 enzyme (New England Biolabs) were added to the annealed PCR products, and the reaction mix was incubated at 37°C for 15 min. The resulting products were then analyzed on a 2% agarose gel to estimate the cleavage rate. For analyzing the CRISPR edits (insertions and deletions), the PCR-amplified products from the targeted regions were subjected to Sanger sequencing. The amplified products were purified and sequenced using BrilliantDye™ v3.1 Terminator Cycle Sequencing Kit (Nimagen) on the 3,500 Genetic Analyzer (Thermo Fisher Scientific). The sequencing results were aligned to the normal sequences using SnapGene (Dotmatics) to estimate the indels or short deletions generated by the dgRNAs. The editing efficiency was quantitated using the DECODR tool (Deconvolution of Complex DNA Repair) (Bloh et al., 2021) or ICE (Inference of CRISPR Edits) (Conant et al., 2022) by comparing the sequencing chromatogram trace files of the edited and the unedited samples.

### Deletion detection by capillary electrophoresis (CE)

The first round of PCR was carried out with target genomic locus-specific primers (Supplementary Table S4) that flank the gRNA binding regions. The forward primers contained a 5′-CAC​TCT​TTC​CCT​ACA​CGA​CGC​TCT​TCC​GAT​CT-3′ tag sequence. The second round of PCRs was performed using a 5′ FAM labeled forward primer 5′-TTT​CCC​TAC​ACG​ACG​CTC​TT-3′ and the same reverse primer used in the first round of PCR. The second round PCR products (∼2 µL) were denatured in deionized formaldehyde and analyzed by capillary electrophoresis using an ABI 3500 Genetic Analyzer (Thermo Fischer Scientific). The peak sizes and peak heights of the amplified products were determined using the Peak Scanner software (Thermo Fisher Scientific).

### Next-generation sequencing to quantitate dgRNA-mediated mutations

Genomic DNA was extracted from transduced BFP+ sorted cells and subjected to a two-step PCR process for mutation quantitation via NGS. Both forward and reverse primers for the first and second rounds of PCR were designed to amplify the target genomic regions, incorporating Illumina adapter sequences. In the first round of PCR, primer sequences included the miRNA genomic site-binding sequence coupled with part of the NGS primer binding site (Forward: 5′-TAC​ACG​ACG​CTC​TTC​CGA​TCT + miRNA genomic locus-specific forward sequence; Reverse: 5′-AGA​CGT​GTG​CTC​TTC​CGA​TCT + miRNA locus-specific reverse sequence). The second round of PCR utilized primers composed of the flow cell binding region (5′-AAT​GAT​ACG​GCG​ACC​ACC​GAG​ATC​TAC​AC) + a unique 6 bp index (NNNNNN)+ and the NGS primer binding site. After secondary PCR, the PCR products with different indices were pooled and purified using a PCR product purification kit (Machery Nagel) and quantitated using Nanodrop and used for sequencing. The prepared libraries were sequenced on the Illumina HiSeq X platform, generating raw data with a read length of 2 bp × 150 bp. Sequencing data was processed to produce Fastq files, which were analyzed using Cas-Analyzer (https://www.rgenome.net/cas-analyzer) (Park et al., 2017).

### Real-time PCR analysis of miRNA expression

Total RNA was extracted from 0.3 to 0.5 million cells using the Nucleozol RNA extraction reagent (Takara Bio). Subsequently, 1 μg RNA was reverse transcribed using the Mir-X™ miRNA First-Strand Synthesis Kit (Takara Bio) as per the manufacturer’s protocol. The quantitative RT-PCR reaction was set up with Go Taq qPCR Master Mix (Promega) using a miRNA-specific forward primer and a common reverse primer provided in the Mir-X™ miRNA First Strand Synthesis Kit (Takara Bio). The miRNA-specific forward primers were designed using miRprimer2 (Busk, 2014). The assay was carried out on QuantStudio 12K Flex Real-Time PCR equipment and software (Thermo Fisher Scientific). Melt/dissociation curves were analyzed before the quantification of miRNAs.

### Western blot

Whole-cell lysates from HUDEP2 and AAVS1-Tet-on-Cas9-HUDEP2 cells were prepared using radioimmunoprecipitation assay (RIPA) buffer (150 mM sodium chloride, 1% Triton X-100, 0.5% sodium deoxycholate, 0.1% SDS, and 50 mM Tris, pH 8), supplemented with Halt Protease Inhibitor Cocktail (Thermo Scientific) and phenylmethanesulfonyl fluoride (PMSF) (Sigma Aldrich). Approximately 15 μg of the lysate was loaded on a 7% sodium dodecyl sulfate-polyacrylamide gel electrophoresis (SDS-PAGE) and analyzed by western blot using primary antibodies, anti-Cas 9 monoclonal antibody (1:5000 dilution) (Cell Signaling Technology), anti-human FANCA (Santa Cruz Biotechnology), anti-human CBR1 (Abclonal), anti-human CTSG (Cell Signaling Technology) and anti-human Actin monoclonal antibody (1: 5000 dilution) (BD Pharmingen) and secondary antibodies, anti-mouse IgG HRP (Cell Signaling Technologies) and anti-rabbit IgG HRP (Invitrogen Corporation Camarillo). The chemiluminescence detection of the protein was performed using the Westar Supernova (Cyanagen) and FluorChemE gel documentation system (Protein Simple).

## Results

### Evaluation of CRISPR/Cas9 sgRNA efficiency in targeting miRNA expression

Previous studies have demonstrated the efficient knockdown of miRNAs through the use of sgRNAs that target crucial functional sequences of miRNAs, such as seed sequences and biogenesis processing sites (Jiang et al., 2014; Chang et al., 2016; Wallace et al., 2016; Huo et al., 2017; Z; Zhang et al., 2018). However, the feasibility of designing efficient sgRNAs within the constrained design space (under 30 nucleotides), which includes these crucial sequences has not been thoroughly investigated for a large number of miRNAs. We analyzed sgRNAs capable of inducing indels at the biogenesis sites and seed sequences of 30 miRNAs (Supplementary Table S1).

**FIGURE 1:**
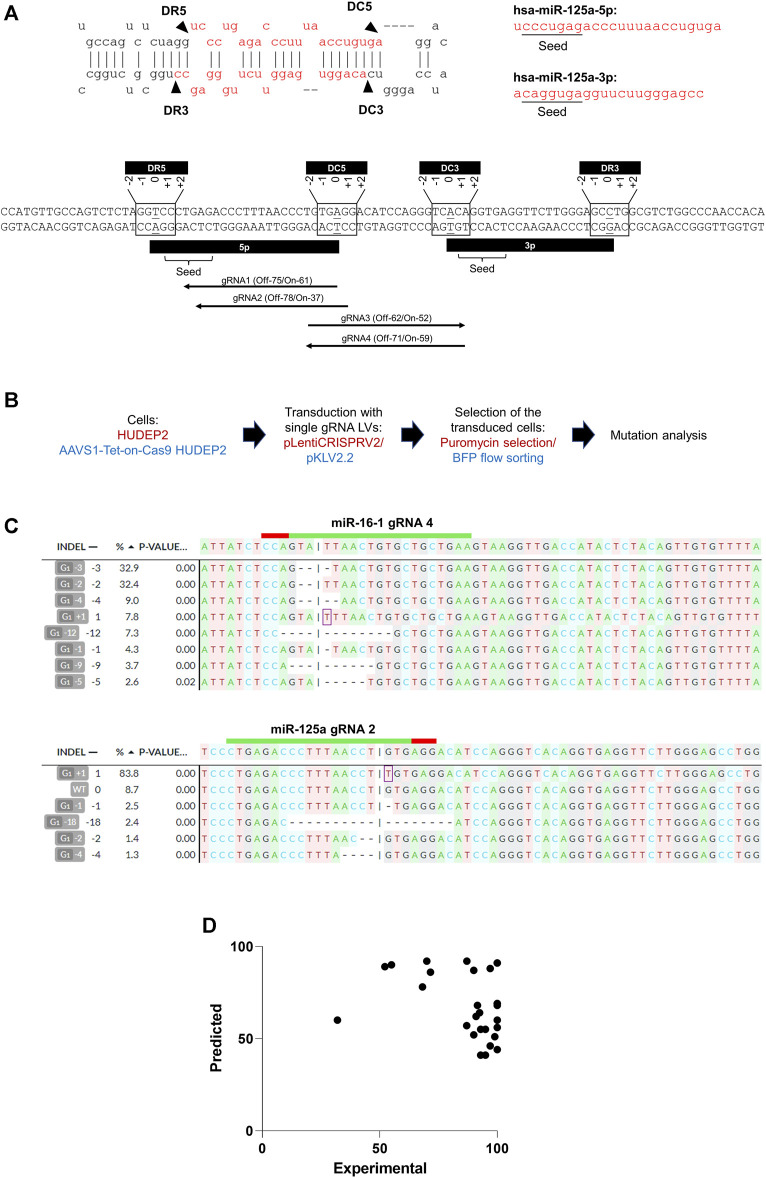
CRISPR/Cas9 editing of miRNA genomic regions using sgRNAs. **(A)** (Upper panel) Secondary structure of a representative miRNA (miR-125a), displaying its biogenesis sites and seed sequences of the 5p and 3p transcripts. DR3 and DR5 represent the 3′ and 5′ Drosha sites, respectively, and DC3 and DC5 represent the 3′ and 5′ of the Dicer sites, respectively. (Lower panel) Genomic regions of the miR-125a highlighting the seed sequences and biogenesis processing sites. The biogenesis sites encompass a 5-base region (−2 to +2) at the 5′ and 3′ ends of the coding regions of the 5p and 3p miRNA transcripts. High off-target score gRNAs with low off-target effects at the seed sequences (gRNA1 and gRNA 2) and the biogenesis sites (gRNA3 targeting DC3 and gRNA4 targeting DC5) are shown. The predicted off-target (“Off”) and on-target (“On”) scores of the gRNAs are indicated. **(B)** Overview of genome editing in HUDEP2 cells using lentivirally expressed sgRNAs. HUDEP2 cells were transduced with the LentiCRISPRV2 lentiviral vector expressing Cas9 and cloned sgRNAs. AAVS1-Tet-On-Cas9 HUDEP2 cells were transduced with pKLV2.2 LV harboring cloned sgRNAs. The transduced cells were selected using puromycin selection (plentiCRISPRV2) or BFP+ flow sorting (pKLV2.2) before mutation analysis. **(C)** Representative results from DECODR analysis of mutations created in two representative miRNA genomic regions (miR-16-1 and miR-125a) by sgRNAs. **(D)** Graph representing the lack of correlation between the predicted and experimental on-target efficiencies of sgRNAs.

The miRNA biogenesis process involves Drosha cleaving at the basal stem junction and Dicer executing cleavage near the loop of the miRNA hairpin structure. Considering the potential variability in the positions of the mutations induced by gRNAs, we included all gRNAs targeting a range of +2 to −2 nucleotides relative to the terminal bases of the 5p and 3p arms (Figure 1A) to encompass all potential gRNAs that could impact miRNA biogenesis. Our analysis identified sgRNAs with high off-target scores (less off-target effects) and high on-target scores (Doench et al., 2016) targeting the 5′ or 3′ Drosha or Dicer sites in 53% (16/30) of the miRNA genomic regions. For seed sequences, effective sgRNAs were found for 30% (18/60) of the miRNAs, taking into account both the 5p and 3p variants from each miRNA genomic region (Figure 1A) (Supplementary Table S1).

The efficacy of gRNAs in inducing specific insertions and deletions (indels) at target sites is largely determined by their on-target editing efficiencies (Xiang et al., 2021). A number of computational tools have been developed for predicting gRNAs with optimal on-target efficiencies (Anthon et al., 2022; Konstantakos et al., 2022). We selected 1 or 2 gRNAs with high off-target scores (score >50) (i.e., low off-target effects) (Hsu et al., 2013), and varying on-target efficiency scores (range: 41–92) (Doench et al., 2016) (Supplementary Table S2) for cloning into lentiviral vectors (Figure 1B). For experiments using the pKLV2.2 lentiviral vector, which does not express Cas9, we generated AAVS1-Tet-On-Cas9 HUDEP2 cells by integrating a tetracycline/doxycycline (Dox)-inducible (Tet-On) Cas9 cassette at the AAVS1 safe harbor site, which is known for sustained and consistent transgene expression (J. R. Smith et al., 2008). A single-cell clone exhibiting robust Cas9 expression post-Dox treatment was expanded to establish a cell line for genome editing (Supplementary Figure S1). Subsequent genome editing experiments conducted with this cell line demonstrated 100% editing efficiency with multiple sgRNAs (Supplementary Table S2) and dgRNAs (Figure 3), validating the homogeneous Cas9 expression facilitating efficient genome editing.

Post-selection (puromycin or flow sorting) of the sgRNA-transduced cells, we conducted the T7EN1 cleavage assay to detect indels at the targeted regions (Supplementary Figure S2) and DECODR analysis (Bloh et al., 2021) to quantitate the mutations (Figure 1C). Although 30% of the guide RNAs (6 out of 20) had lower predicted on-target efficiency scores (<50) (Haeussler et al., 2016), they demonstrated high experimental editing efficiencies, with an average efficiency of 91.93% ± 16.033% (Supplementary Table S2). To further investigate, we transduced HUDEP2 cells with 12 sgRNAs, cloned into the pL-CRISPR.EFS.GFP gRNA and Cas9 expression vector, to target the duplicated human γ globin promoter sequences and compared the predicted and experimental on-target efficiencies (Supplementary Table S2). The collective data from 32 sgRNAs demonstrated a mean editing efficiency of 92 ± 16.03 (ranging from 32% to 100%) although their on-target efficiencies ranged from 41 to 92 (Supplementary Table S2) (Figure 1D). Our findings suggest that a significant number of miRNAs may not be amenable to editing at the biogenesis sites and seed sequences with sgRNAs with high on-target and off-target scores. Additionally, we observed a lack of correlation between the predicted and actual on-target efficiencies of lentivirally expressed gRNAs (Figure 1D), suggesting the possibility of including gRNAs with low on-target efficiencies when employing lentiviral vectors for gRNA expression.

### Optimization of efficient dgRNA-mediated deletions of miRNAs in HUDEP2 cells

To simplify the generation of dgRNA expression vectors (Nath et al., 2021) and prevent intra-plasmid recombination (Vidigal and Ventura, 2015), we employed a single lentiviral plasmid featuring two distinct RNA polymerase III promoters and unique restriction enzyme sites for sequential cloning of gRNAs (Supplementary Figure S3A). We assessed the gene editing efficiencies of three gRNA expression promoters [human U6 (hU6), mouse U6 (mU6), and human 7SK (h7SK)] by cloning two gRNAs with varied on-target efficiency scores (scores of 6 and 73) under each promoter (Supplementary Figure S3B). Editing efficiency analysis in transduced HUDEP2 cells showed no significant differences among the promoters (Supplementary Figures S4A, B). Consequently, for our experiments to create short genomic deletions, we used the pKLV2.2 vector with h7SK and hU6 promoters for the expression of the dgRNAs.

We designed 33 dgRNA pairs to target 23 miRNA genomic regions. This included 10 miRNAs targeted by two dgRNA pairs and 13 miRNAs targeted by a single pair of dgRNAs (Supplementary Table S3). The selection of these miRNAs was based on their differential expression during erythroid differentiation (Supplementary Figure S5). The designed gRNA pairs had low off-target effects, while their on-target efficiency scores varied (CRISPOR Doench scores range: 31–78) (Doench et al., 2016). They were designed to flank 5p or 3p arms of the targeted miRNAs (Supplementary Figure S6) and to bind either to the same or opposite DNA strands. Their PAM offsets (the distance between the PAMs of the dgRNAs) ranged from 36 bp to 79 bp and gRNA offsets (the distance between the dgRNAs) ranged from 2 bp to 101 bp (Supplementary Table S3). Two pairs contained overlapping gRNAs (gRNAs 3 and 4 for miR-15 and gRNAs 1 and 2 for miR-223) (Supplementary Table S3) (Supplementary Figure S7A).

**FIGURE 2:**
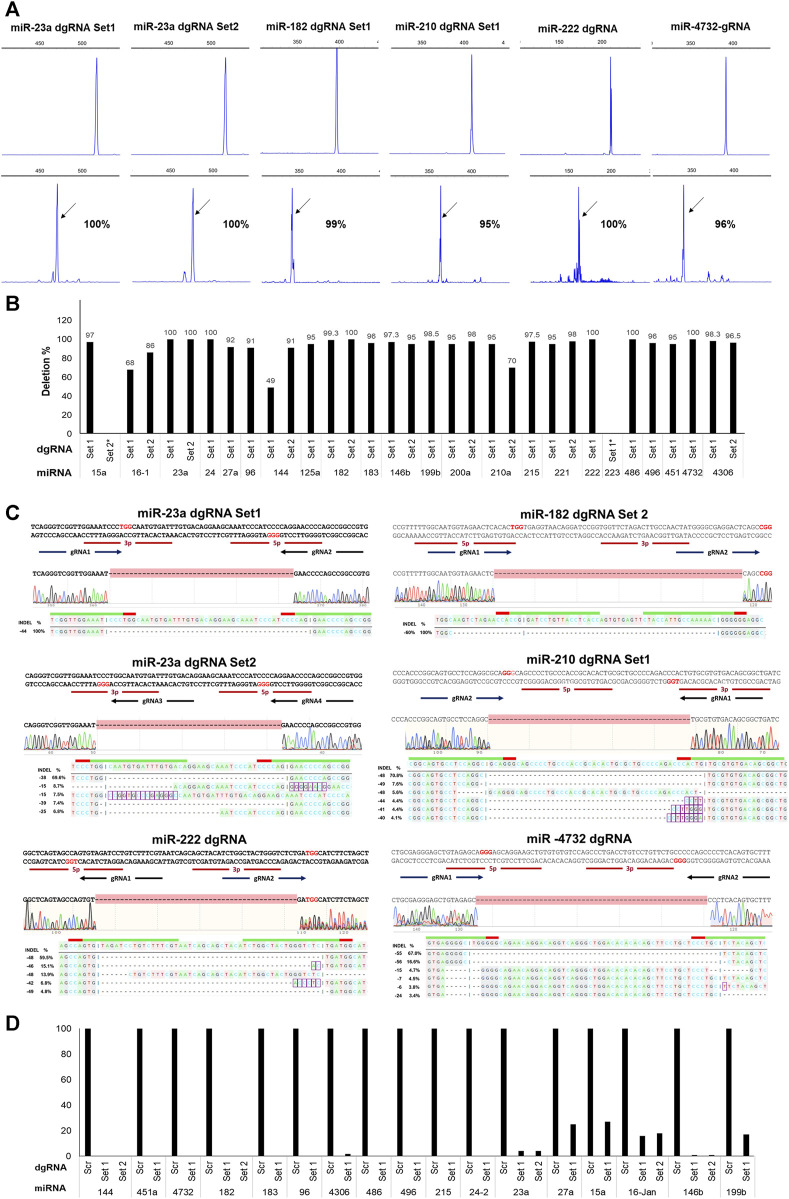
Efficient deletion formation in HUDEP2 cells using dgRNA expressing lentiviral vectors. **(A)** Representative FL-PCR-CE results of 6 miRNA genomic regions targeted by dgRNAs. The upper panel displays fragments formed from the unedited cells and the lower panel shows fragments from the respective edited cells. The arrows indicate specific fragments resulting from deletions. The corresponding percentages of deletions are also shown. **(B)** Graph depicting the deletion percentages detected by FL-PCR-CE in different miRNA regions **(C)** Sanger sequencing alignment and DECODR mutation analysis of the PCR products showing deletions in the targeted regions. **(D)** Real-time PCR analysis results showing a significant reduction in expression levels of targeted miRNAs in the knockout cells.

After AAVS1-Tet-On-Cas9 HUDEP2 cells were transduced with individual dgRNA lentiviruses, BFP+ cells were flow-sorted and then analyzed for mutations at the targeted regions. Agarose gel electrophoresis (AGE) detected deletion formation for 30 out of 33 dgRNA pairs, with complete deletions observed only in 5 pairs (Supplementary Figure S8). Capillary electrophoresis of fluorescently labeled PCR products (FL-PCR-CE) quantified deletion formation, revealing a higher percentage of deletions (mean ± SD: 87.5578 ± 24.66; range: 0%–100%) compared to AGE, with 27 pairs achieving ≥90% deletion efficiency (mean ± SD: 96.90 ± 2.705), out of which 7 pairs demonstrating 100% deletion efficiency (Figure 2A) (Figure 2B) (Supplementary Table S3). Sanger sequencing followed by DECODR analysis (Bloh et al., 2021) of the targeted regions of 25 samples showed a strong correlation with the CE results (Figure 2C) (Supplementary Table S3). We also performed next-generation sequencing of the amplified products from 10 miRNA genomic regions targeted by dgRNAs, and the data confirmed high rate of deletion formation in the targeted miRNAs (Supplementary Figure S9). The overlapping gRNA pairs predominantly resulted in indels rather than deletions (Supplementary Figure S7B). Excluding these pairs, the overall percentage of deletions was 93.206 ± 11.17 (Supplementary Table S3). Real-time PCR analysis of the 17 targeted miRNAs, which had significant expression levels in HUDEP2 cells, revealed reductions in their expression levels (mean: 94.75 ± 8.67; range: 73%–100%) (Figure 2D).

Despite low BFP+ cell percentages (ranging from 9% to 30%) in 13 dgRNA transductions, we observed high deletion formation in the flow-sorted BFP+ cells (mean ± SD: 99.08 ± 2.055; range: 93%–100%), demonstrating the efficacy of short genomic deletion formation even with low lentiviral integration of dgRNAs. Given the rapid differentiation of certain human primary cells, such as hematopoietic progenitors, in culture, quickly achieving miRNA deletions is crucial for studying the phenotypic effects of miRNA knockouts in undifferentiated cells. Therefore, we assessed the deletion formation ability of 10 dgRNA pairs that showed very high deletion percentages (mean ± SD: 97 ± 2.738; range: 93%–100%) 5 days post-transduction and observed similar high deletion percentages (mean ± SD: 99.3 ± 1.417; range: 96%–100%). Efficient deletion formation of individual miRNAs was obtained gRNA offset of up to 71 bp. Overall, our findings highlight the effectiveness of dgRNA design, the selection of lentiviral vectors for their expression, and the homogeneous expression of Cas9 for rapid and efficient generation of short genomic deletions.

### Evaluating the efficacy of lentiviral dgRNA-mediated miRNA deletions in iPSCs

Induced pluripotent stem cells (iPSCs), reprogrammed from somatic cells (Takahashi et al., 2007), possess the unique capability to remain undifferentiated and can be directed to differentiate into various cell lineages, including hematopoietic progenitors (Sturgeon et al., 2014; Netsrithong et al., 2020; Dannenmann and Skokowa, 2022). Inducing miRNA deletions in undifferentiated iPSCs and during hematopoietic differentiation provides an opportunity to explore the roles of specific miRNAs in different hematopoietic cell lineages, such as erythroid cells. We aimed to assess the efficacy of lentiviral dgRNA-mediated miRNA deletions in the AAVS1-Tet-On-Cas9 iPSC line, where the Dox-inducible Cas9 cassette is integrated at the AAVS1 site (Thamodaran et al., 2022). We focused on deleting 7 miRNAs from the miR-144/451, miR-183/96/182, and miR-23a/27a/24-2 clusters. Notably, the miRNAs from the miR-144/451 and miR-183/96/182 clusters are the most upregulated miRNAs during human erythropoiesis (Nath et al., 2021). The members of the miR-144/451 cluster are involved in erythroid maturation, and those of miR-23a/27a/24-2 clusters are associated with the regulation of various aspects of hematopoietic stem cell differentiation and lineage commitment (Kurkewich et al., 2017) and lineage development (Cho et al., 2016).

**FIGURE 3:**
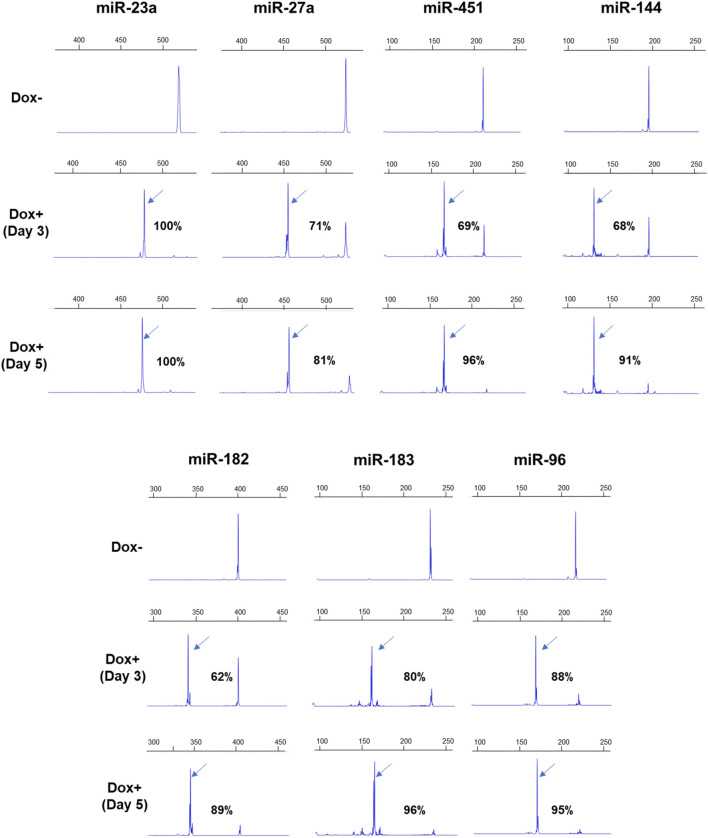
FL-PCR-CE results of 7 miRNA genomic regions targeted in iPSCs by dgRNAs. The upper panel displays the electropherograms of PCR fragments formed from the targeted regions in the unedited iPSCs, cultured without Dox (-Dox). The middle and lower panels show electropherograms of fragments derived from edited regions after 3 (Day 3) and 5 (Day 5) days of Dox supplementation, respectively. The arrows indicate specific fragments resulting from deletions. The corresponding percentages of deletions are also shown.

The AAVS1-Tet-On-Cas9 iPSCs were transduced with pLKV2.2 lentiviruses expressing dgRNAs, and the flow-sorted BFP+ cells were cultured with Dox. Deletion formation was analyzed at 3 and 5 days after Dox supplementation by AGE (Supplementary Figure S10) and FL-PCR-CE (Figure 3). FL-PCR-CE detected higher percentages of deletions than AGE, consistent with observations in HUDEP2 cells. On day 3, deletion percentages ranged from 62% to 96% (mean ± SD: 76.29 ± 11.30) as determined by CE, and increased to 81%–100% (mean ± SD: 92.57 ± 5.78) (Figure 3) by day 5. Notably, significant cell death (>90% cells) was observed in cultures with dgRNAs targeting miR27a and miR23a, suggesting the critical role of these miRNAs in the survival of pluripotent stem cells. These results emphasize the efficacy of lentiviral dgRNA-mediated miRNA deletion in iPSCs, showing great potential for investigating the specific roles of miRNAs in iPSC maintenance and differentiation.

### Analyzing the impact of miRNA deletions on erythroid differentiation in HUDEP2 cells

**FIGURE 4:**
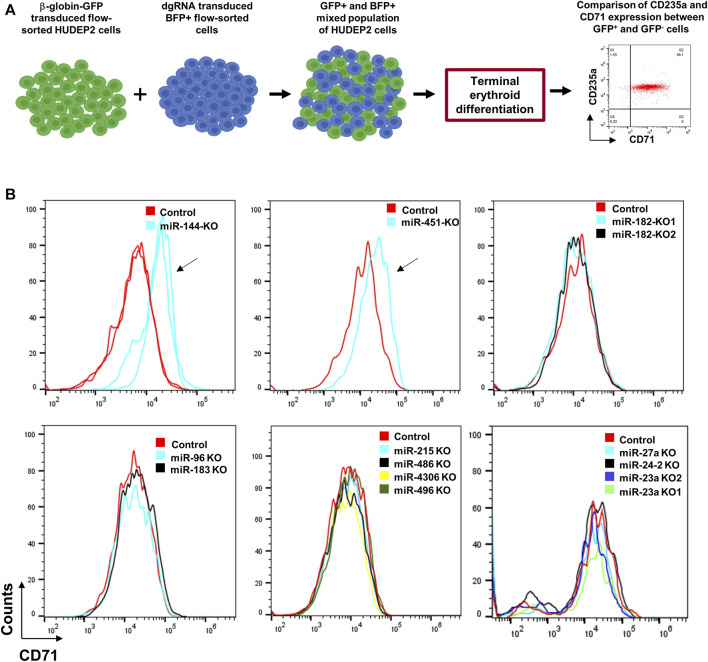
Differentiation of miRNA-knocked out HUDEP2 cells. **(A)** Experimental setup to study the effect of miRNA deletions on differentiation of HUDEP2 cells. The dgRNA transduced BFP+ HUDEP2 cells were mixed with HUDEP2 cells transduced with β-globin promoter-GFP lentiviral vector. The mixed population was then subjected to terminal differentiation and CD71 and CD235a expressions were compared in the GFP- and GFP+ cells. **(B)** Flow cytometry data showing CD71 expression in the terminally differentiated unedited and edited cells. The miRNA knockouts included miR-182, miR-183, miR-96, miR-144, miR-451a, miR-4732, miR-4306, miR-215, miR-486, miR-496, miR-23a, miR-24-2 and miR-27a. The arrows are used to indicate the difference in differentiation kinetics in miR-144 and miR-451 knockout HUDEP2 cells compared to the control cells. KO1 and KO2 refer to knockouts using dgRNA set 1 and set 2, respectively.

To assess the effectiveness of our miRNA deletion strategy in elucidating the role of miRNAs in erythroid differentiation, we utilized HUDEP2 cells, known for their differentiation capacity into late-stage erythroid cells. Among the tested miRNAs for deletion formation, miR-144 and miR-451 are known to significantly influence erythroid differentiation (Rasmussen et al., 2010; Kim et al., 2015). AAVS1-Tet-On-Cas9 HUDEP2 cells transduced with dgRNAs targeting miR-144, miR-182, miR-183, miR-451, miR-4732, miR-96, miR-4306, miR-215, miR-486, miR-496, miR-27a, miR-23a, and miR-24-2 were used for the experiment (Figure 4A). We examined CD71 and CD235a expression, two erythroid surface markers, to understand miRNA knockout impacts on erythroid differentiation kinetics. Undifferentiated HUDEP2 cells co-express CD71 and CD235a surface markers, but during differentiation CD71 expression undergoes a decline, whereas CD235a expression remains stable throughout differentiation. Defects in erythroid differentiation can be identified by observing a slower or no decrease in CD71 expression, indicating potential disruptions in the maturation of erythroid cells.

Due to the gradual decline in BFP expression during differentiation, likely resulting from transgene silencing by the PGK (phosphoglycerate kinase 1) promoter, analyzing differentiation kinetics between BFP+ and BFP- cells in a mixed population proved challenging. To address this issue, we combined the dgRNA-transduced cells with HUDEP2 cells transduced with a lentiviral vector, which expresses GFP under the β-globin promoter (Figure 4A). Notably, the β-globin promoter is renowned for its strong transcriptional activity during erythroid differentiation (Bagchi et al., 2022). Upon subjecting the mixed cell population to terminal differentiation, we analyzed the expression of CD71 and CD235a in both GFP+ (β-globin promoter-transduced) and GFP- (dgRNA-transduced) cells. Knockout of miR-144 and miR-451 led to arrested differentiation (Figure 4B), whereas other miRNA knockouts did not significantly alter differentiation kinetics compared to control cells, despite their upregulation during erythroid differentiation (Figure 4B).

### Efficacy of lentiviral dgRNAs in inducing deletions across diverse genomic regions in multiple cell types

**FIGURE 5:**
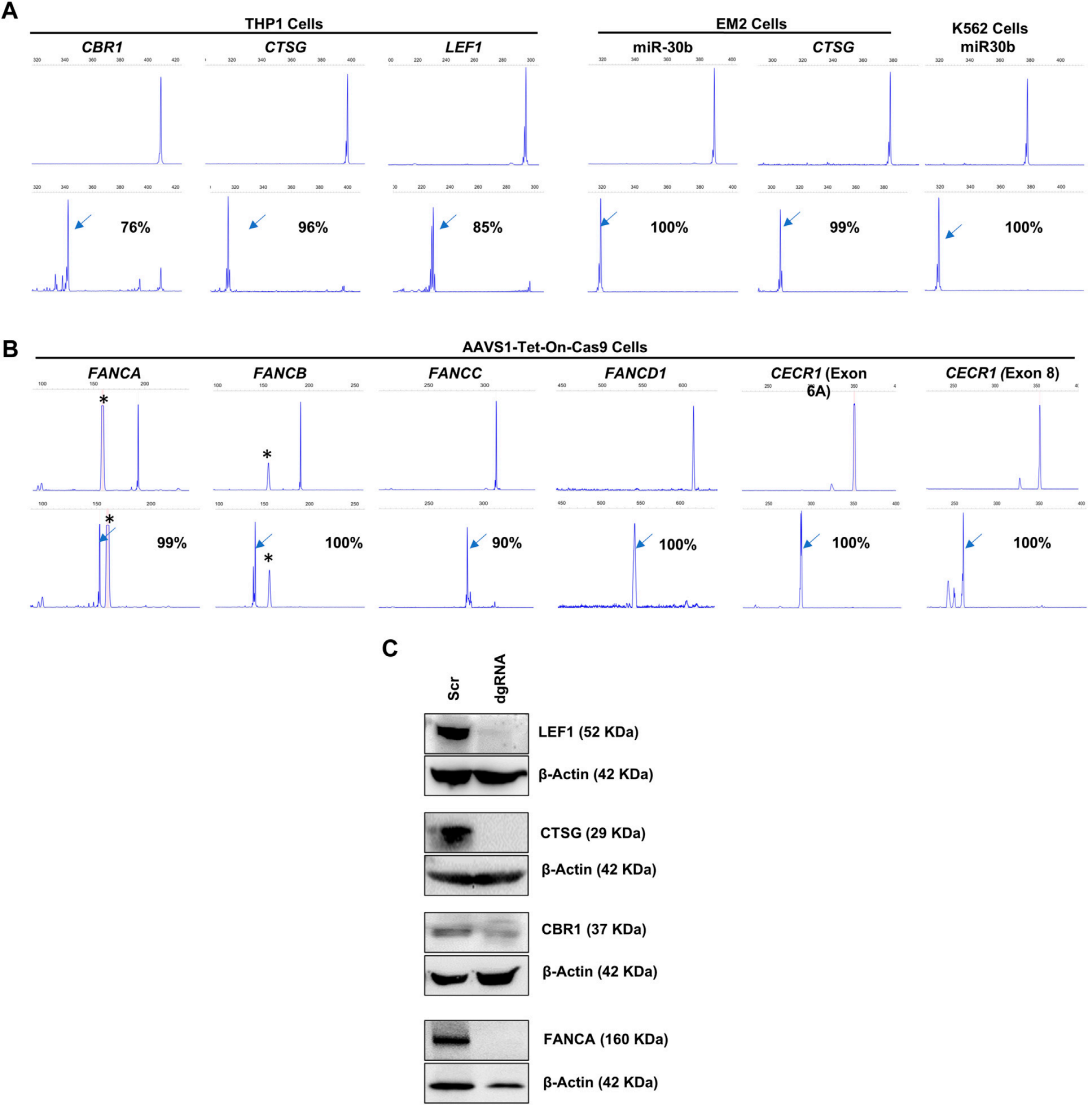
FL-PCR-CE analysis of dgRNA mediated deletion formation in protein-coding genes and intergenic regions. **(A)** Targeting *CBR1* (exon 1), *CTSG* (exon 1) and *LEF1* (exon 4) in THP1-Cas9 cells and *CTSG* (exon 1) in EM2-Cas9 cells, using dgRNAs Targeting miR-30b in EM2-Cas9 and K562-Cas9 cells are also shown. **(B)** Targeting *FANCA* (exon 2), *FANCB* (exon 1), *FANCC* (exon 5), *FANCD1* (exon 10) and CECR1 (Exon 6A and Exon 8) with dgRNAs in AAVS1-Tet-On-Cas9 iPSCs. The upper panels of **(A**,**B)** display fragments formed from the unedited cells and the lower panel shows fragments from the edited cells. The arrows indicate specific fragments resulting from deletions. The corresponding percentages of deletions are shown. Asterisks (*) indicate non-specific peaks present in the PCR products from both edited and unedited cells. **(C)** Immunoblot analysis to assess protein expression from the dgRNA-targeted genes.

Following the successful induction of deletions in the miRNA genomic regions, we assessed the capacity of lentivirally transduced dgRNAs to create deletions at other genomic regions, including both protein-coding genes and intergenic regions (Figures 5A, B). We first developed THP1, K562, and EM2 cell lines with stable Cas9 expression through lentiviral transduction. We targeted *LEF1*, *CTSG*, and *CBR1* genes in THP1-Cas9 cells, miR-30b and *CSTG* in EM2-Cas9 cells, and miR-30b in K562-Cas9 cells (Figure 5A). Additionally, we targeted the protein-coding genes *FANCA*, *FANCB*, *FANCC,* and *FANCD1* in AAVS1-Tet-On-Cas9 iPSCs (Figure 5B). We also targeted the DNase I hypersensitive sites HS1, HS2, HS3, and HS4, which constitute transcriptional enhancer elements in the β-globin cluster in AAVS1-Tet-On-HUDEP2 cells. The dgRNA-transduced cells exhibited 76%–100% deletion formation (mean + SD = 95.4 + 7.5) in the target regions within a week across all cell types (Figure 5A). Immunoblot analysis of four targeted proteins revealed a notable decrease in expression, with three exhibiting a complete absence of expression (Figure 5C). In summary, our research effectively showcased the efficiency of lentivirally transduced dgRNAs in producing deletions, extending beyond miRNA genomic regions to include both protein-coding genes and intergenic regions. These findings underscore the potential of our lentiviral approach for efficient genome editing across diverse genomic and cellular contexts.

## Discussion

Understanding the functional roles of miRNAs is crucial for unraveling the complexities of gene regulation that govern various cellular processes. The extensive impact of miRNAs has been implicated in their dysregulation associated with various diseases, including cancer, cardiovascular disorders, and neurological conditions (Calin and Croce, 2006). For miRNA loss-of-function studies, selectively suppressing specific miRNAs, while minimizing the impact on similar off-target miRNAs, is critical. In this study, we introduce a novel approach utilizing CRISPR/Cas9 and dgRNAs for the efficient generation of miRNA deletions within a short timeframe (approximately 5 days). This method achieves highly efficient knockdown (>90%) of targeted miRNAs with single-copy lentiviral integration of dgRNAs in human cell lines.

Traditional miRNA inhibitory approaches, such as antisense oligonucleotides (Hutvágner et al., 2004; Krützfeldt et al., 2006) and sponges/decoys (Ebert et al., 2007; Ebert and Sharp, 2010), effectively induce miRNA loss of function but lack specificity when targeting miRNAs with similar seed sequences. Conversely, sgRNA-based CRISPR/Cas9 systems offer greater specificity but are limited by a design space of about 25 bases, encompassing the miRNA biogenesis sites and seed sequences. Our CRISPR/Cas9 method with dgRNAs shows exceptional efficiency in miRNA knockout and allows more flexible gRNA design, enabling targeting within the entire miRNA region, beyond just the biogenesis processing sites or seed sequences. Targeting either the 3p or 5p genomic region of the miRNAs, which form their double-stranded stem structure, impacts the expression of the other region, thus broadening the design scope for specific miRNA modulation. The efficacy of this method is partly due to the robustness of the lentiviral dual-gRNA approach and the use of cell lines with integrated Cas9, which ensures consistent expression of the genes with minimal transgene silencing (J. R. Smith et al., 2008). The use of Dox-inducible Cas9 expression allows for regulated and stage-specific miRNA knockout in various cell types, including undifferentiated stem cells, progenitors, and differentiated cells. This versatility provides valuable insights into miRNA roles across different cell types. While previous studies have explored dgRNAs for miRNA knockouts, they often show low efficiency, necessitating single-cell sorting to create cell lines with miRNA knockouts (Ho et al., 2015; Liu et al., 2016; Yan et al., 2019; Hong et al., 2020). Our study found that following the establishment of Cas9-expressing cell lines (approximately 1 month), the transduction of dgRNAs and generation of deletions could be achieved in under a week.

MicroRNAs (miRNAs) are pivotal in regulating transcription signals, growth factors, and epigenetic cell control (Zahnow and Baylin, 2010; Gruber and Zavolan, 2013; Pagano et al., 2013). This study employs a dgRNA-mediated approach for miRNA deletion in two human cell lines: HUDEP2, used in erythropoiesis research, and induced pluripotent stem cells (iPSCs), valuable for studying cellular differentiation and disease mechanisms. Our findings in HUDEP2 cells show that the knockout of miR-144 and miR-451a significantly alters erythroid differentiation. By efficiently knocking out the target miRNAs, we discovered that the loss of miR-27a and miR-23a expression led to pronounced cell death, indicating the importance of these miRNAs for the survival of pluripotent stem cells. Dox-inducible miRNA knockout in hematopoietic stem and progenitor cells (HSPCs) and erythroid cells will enhance our understanding of the function of these miRNAs in various cellular contexts.

In our systematic assessment of the dgRNA system, we evaluated parameters including on-target gRNA efficiencies and the orientation and distance between gRNAs, to discern their effects on deletion efficiency. We observed over 80% efficiency in deletion formation with gRNA offsets ranging from 2 to 101 bases. Interestingly, the orientation and binding position of the gRNAs, whether on the same or opposite strands, did not significantly affect the efficiency of deletion formation. While AGE was insufficient in detecting substantial deletion formation in our targets, FL-PCR-CE, analyzing single-stranded PCR products, successfully identified a high rate of deletions in most targeted miRNAs. This discrepancy is likely due to the formation of heteroduplexes between PCR products with different sequence alterations at the deletion breakpoints. Sanger sequencing of the targeted regions corroborated the presence of various deletion breakpoints, supporting the heteroduplex formation hypothesis. FL-PCR-CE proved to be a highly sensitive method for detecting short deletions caused by dgRNAs, presenting benefits of speed, cost-efficiency, and ease of use, compared to next-generation sequencing (NGS) methods. Among the 31 dgRNAs that showed deletion formation in HUDEP2 cells, 3 (10%) exhibited deletion percentages below 80% (Supplementary Table S3). For these cells, single-cell sorting might be necessary to isolate populations with higher deletion percentages. The high efficiency of genomic deletions achieved by dgRNAs was further substantiated through protein-coding sequence editing and subsequent protein expression analysis. We also enhanced the dgRNA lentiviral vectors by creating several variants. These modifications included substituting BFP with green (GFP) and red fluorescence proteins (RFP) for compatibility with different fluorescence markers or antibodies when multi-fluorescence experiments are performed (Supplementary Figure S11A), and replacing the PGK promoter with the EF1α and MND promoters to enhance fluorescence protein expression in stem cells (Supplementary Figure S11B). Additionally, we substituted short stuffer sequences with longer ones to ensure complete digestion of the backbone before gRNA cloning (Supplementary Figure S11C).

Future research will benefit from experiments aimed at determining the maximum effective distance between guide RNAs (gRNAs) for efficient miRNA deletion. This is crucial for developing protocols to simultaneously delete multiple miRNAs within a cluster. Targeting individual miRNAs in a cluster might result in limited phenotypic changes, but the collective downregulation of all members in the cluster can have significant effects, given their cooperative amplification of regulatory impacts (Dylla and Jedlicka, 2013). The dgRNA strategy proposed here can facilitate the creation of CRISPR/gRNA libraries. These libraries could be instrumental for high-throughput screening of all known miRNAs, thereby serving as a potent tool in identifying miRNAs associated with specific biological processes and elucidating their complex roles in disease pathogenesis, cellular differentiation, and other key biological events. However, before conducting such high-throughput assays, it is essential to evaluate the off-target effects of designed gRNAs. Employing advanced algorithms to select gRNAs with minimal off-target consequences (Concordet and Haeussler, 2018; Dhanjal et al., 2020; Trivedi et al., 2020; Dhanjal et al., 2020; Niu et al., 2021; Z.-R; Zhang and Jiang, 2022) is essential for maintaining the specificity of the CRISPR/Cas9 system. Additionally, the application of Cas9 variants known for reduced off-target activity, such as enhanced S. pyogenes Cas9 (eSpCas9) (Slaymaker et al., 2016) and high-fidelity SpCas9-HF1 (P. H. Smith et al., 2016), could significantly enhance the precision of CRISPR/Cas9-mediated miRNA editing. Exploring other Cas variants, like Cas12a (Ma et al., 2022) and CasX (Tsuchida et al., 2022), may offer further advancements in the disruption of miRNA genomic regions.

## Data Availability

Publicly available datasets were analyzed in this study. This data can be found here: The small RNA sequencing discussed in this manuscript has been deposited in NCBI’s Gene Expression Omnibus (GEO) and is accessible through GEO Series accession number GSE185685.
