# Supplementary material for: Efficient deletion of microRNAs using CRISPR/Cas9 with dual guide RNAs
Source: Front Mol Biosci. 2024 Apr 2;10:1295507. doi: 10.3389/fmolb.2023.1295507 (PMC11020096; doi:10.3389/fmolb.2023.1295507)
Supplement: Supplementary file 1 [file DataSheet1.PDF]

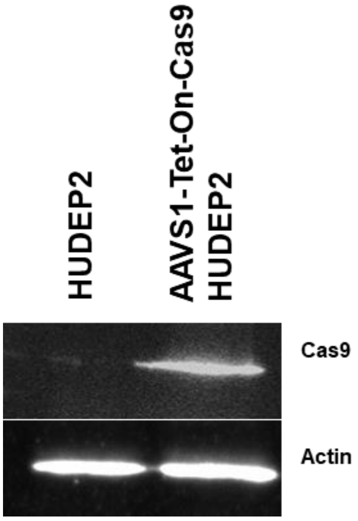

Supplementary Figure S1. Immunoblot analysis of Cas9 expression in AAVS1-Tet-On-Cas9 HUDEP2 cell line. Whole-cell extract from normal HUDEP2 cells served as a negative control.

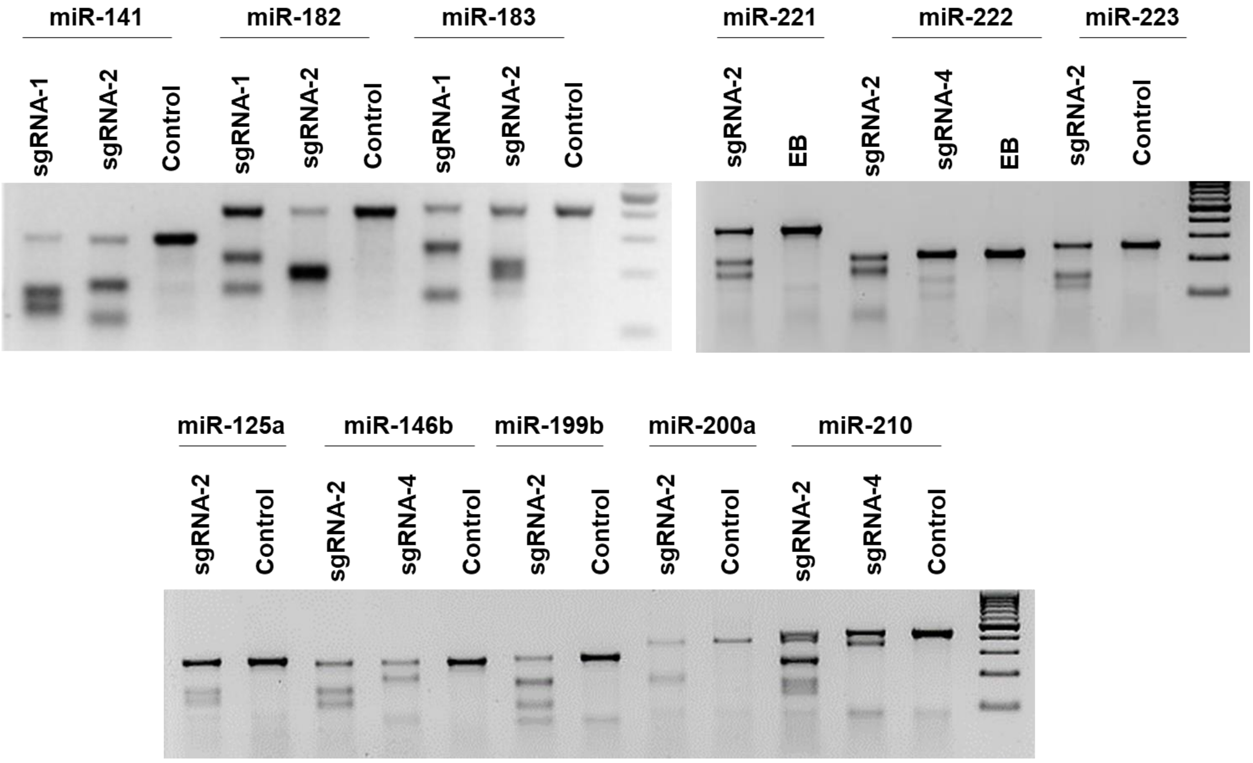

Supplementary Figure S2. T7EN1 analysis of PCR amplified products from the miRNAs targeted with sgRNAs cloned in LentiCRISPR V2 and pLKV2.2 lentiviral vectors. The cells transduced with lentiviruses generated with empty backbone plasmid were used as controls.

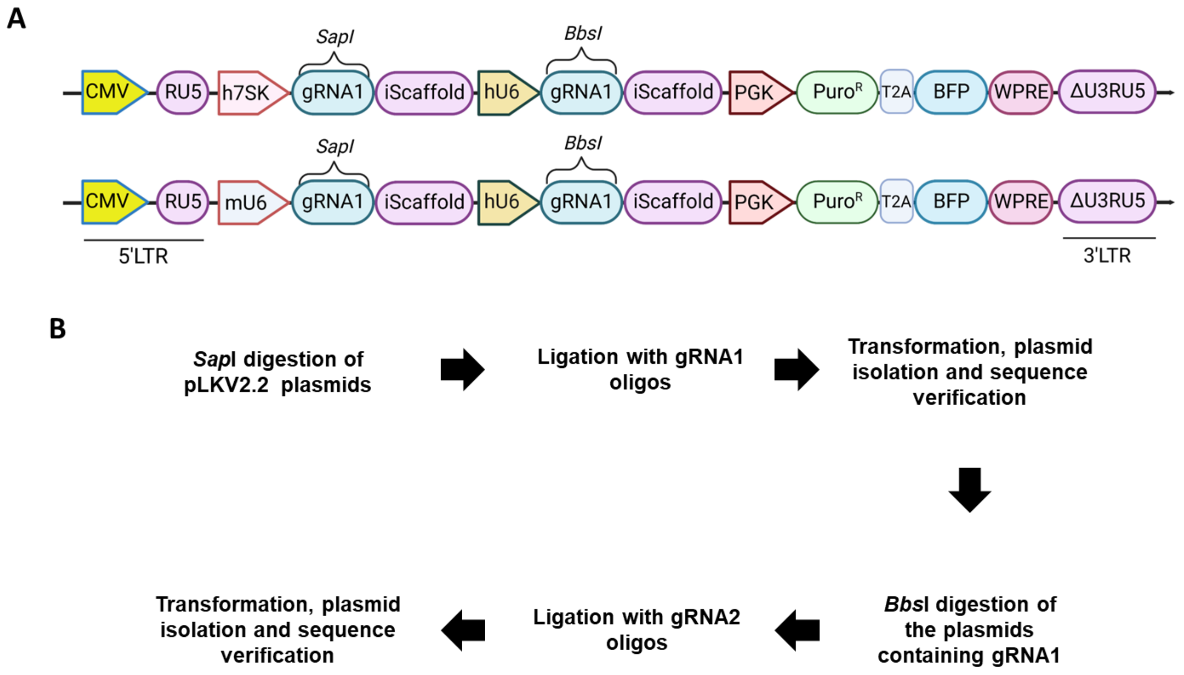

Supplementary Figure S3. The cloning strategy of dgRNAs in the pLKV2.2 vector. (**A**). Schematic of pKLV2.2 dgRNA lentiviral expression vectors (Tzelepis et al., 2016), which has improved tracrRNA scaffold (iScaffold) and different RNA polymerase **III** promoters for dgRNA expression. Key elements include 5' and 3' long terminal repeats (LTRs), human U6 promoter (hU6), human 7SK promoter (h7SK), gRNA cloning sites with *Bbs*I and *Sap*I, mouse Pgk1 promoter (PGK), puromycin resistant gene (PuroR), thosea asigna virus 2A peptide (T2A), blue fluorescent protein gene (BFP), and woodchuck hepatitis virus posttranscriptional regulatory element (WPRE). (B) The schematic representation of cloning of dgRNAs in pKLV2.2 vectors.

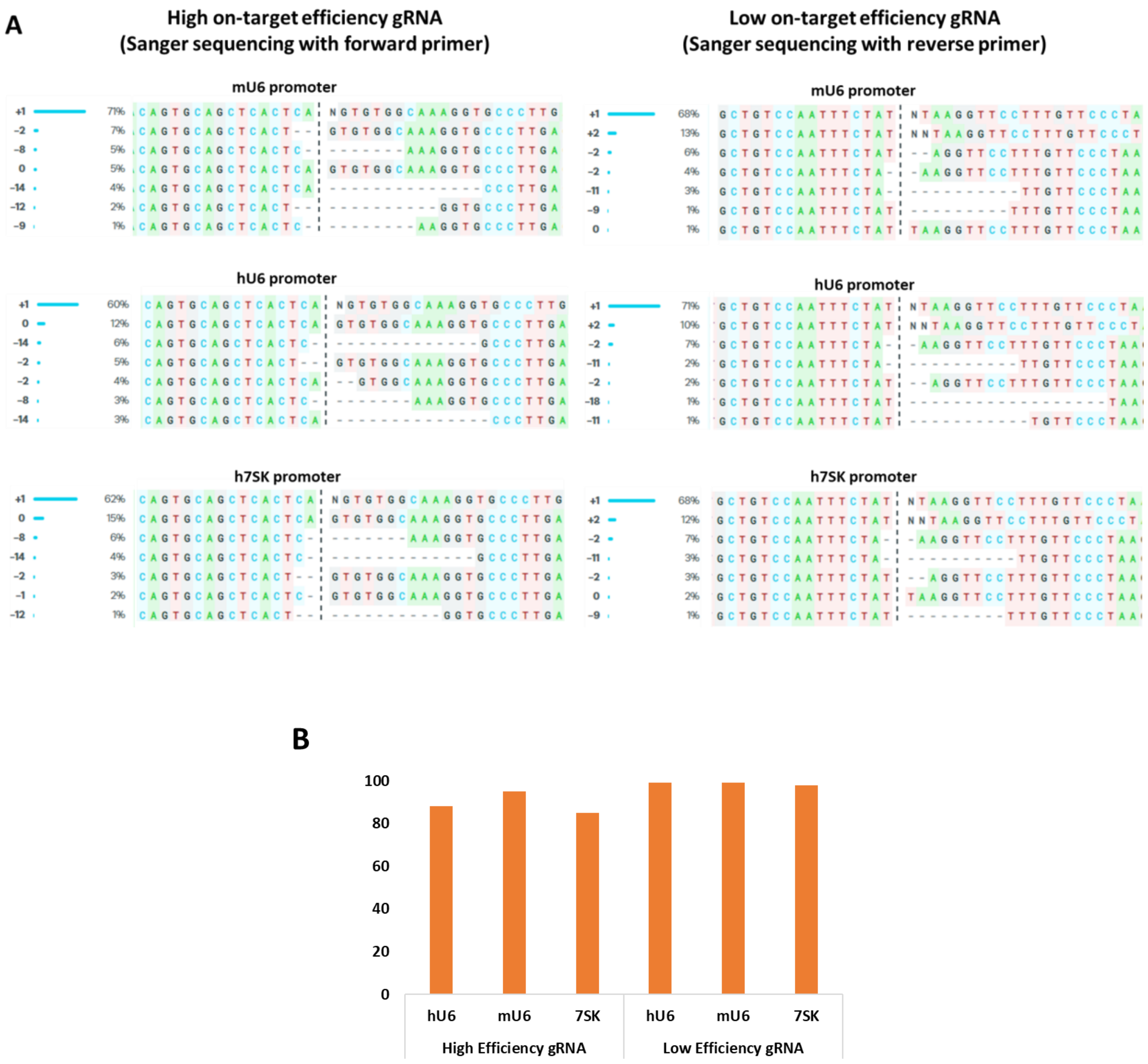

Supplementary Figure S4. (**A**) ICE analysis of Sanger sequencing traces obtained with the PCR-amplified products of the targeted regions to compare the efficiency of hU6, mU6, and hSK gRNA expression promoters for two different gRNAs, one with a low on-target efficiency score and the other with a high on-target efficiency score (**B**) Graphical representation of the experimental editing efficiencies by the low and high on-target efficiency gRNAs by three different gRNA expression promoters.

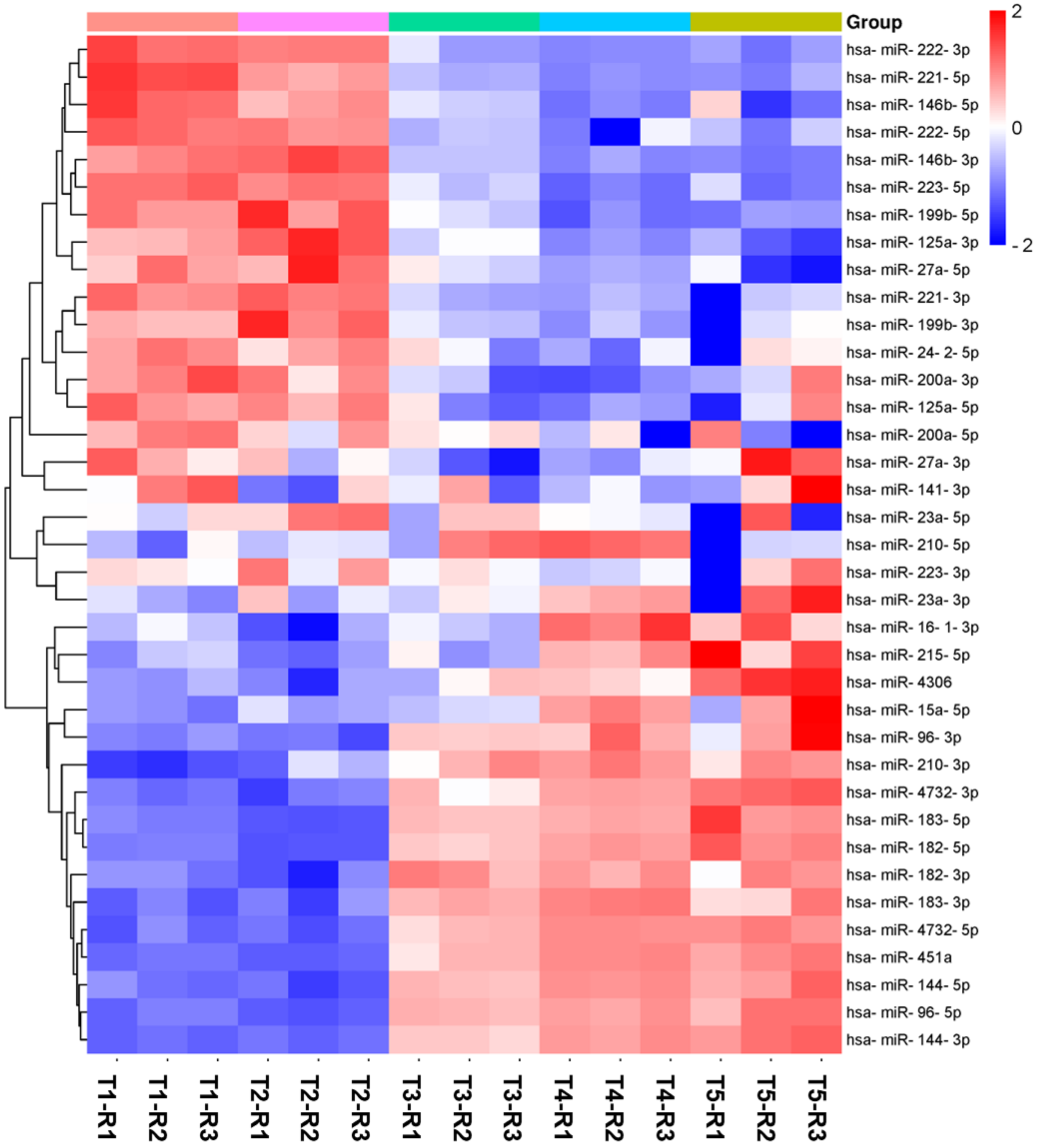

Supplementary Figure S5. Differential expression of miRNAs at different stages of ex-vivo erythropoiesis. Small RNA sequencing carried out in the cells at 5 different time points (T1, T2, T3, T4, and T5) of *ex vivo* erythroid differentiation. T1 represents undifferentiated CD34+ hematopoietic progenitor cells and T2, T3, T4, and T5 represent the erythroid cells from different stages of differentiation. R1, R2, and R3 denote replicates of each time point.

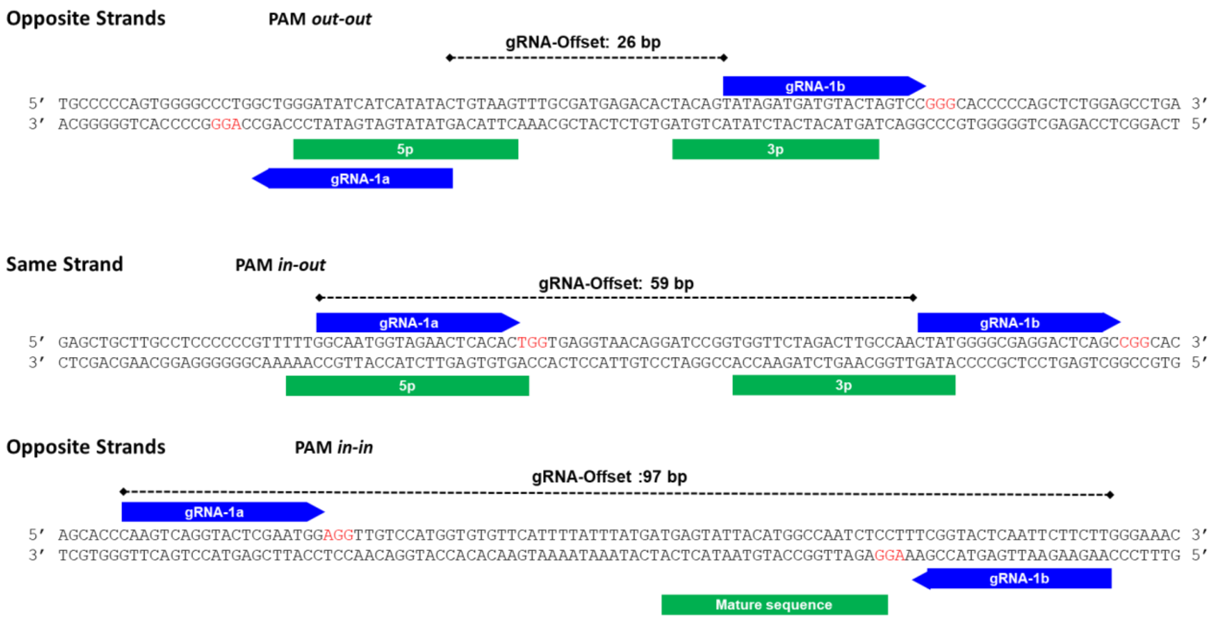

Supplementary Figure S6. Representative figures showing different PAM orientations (out-out, in-out, out-in, or in-in) and gRNA offsets of the dgRNAs designed for various miRNA genomic regions. The gRNA-offset refers to the distance between the 5’ ends of the dgRNAs.

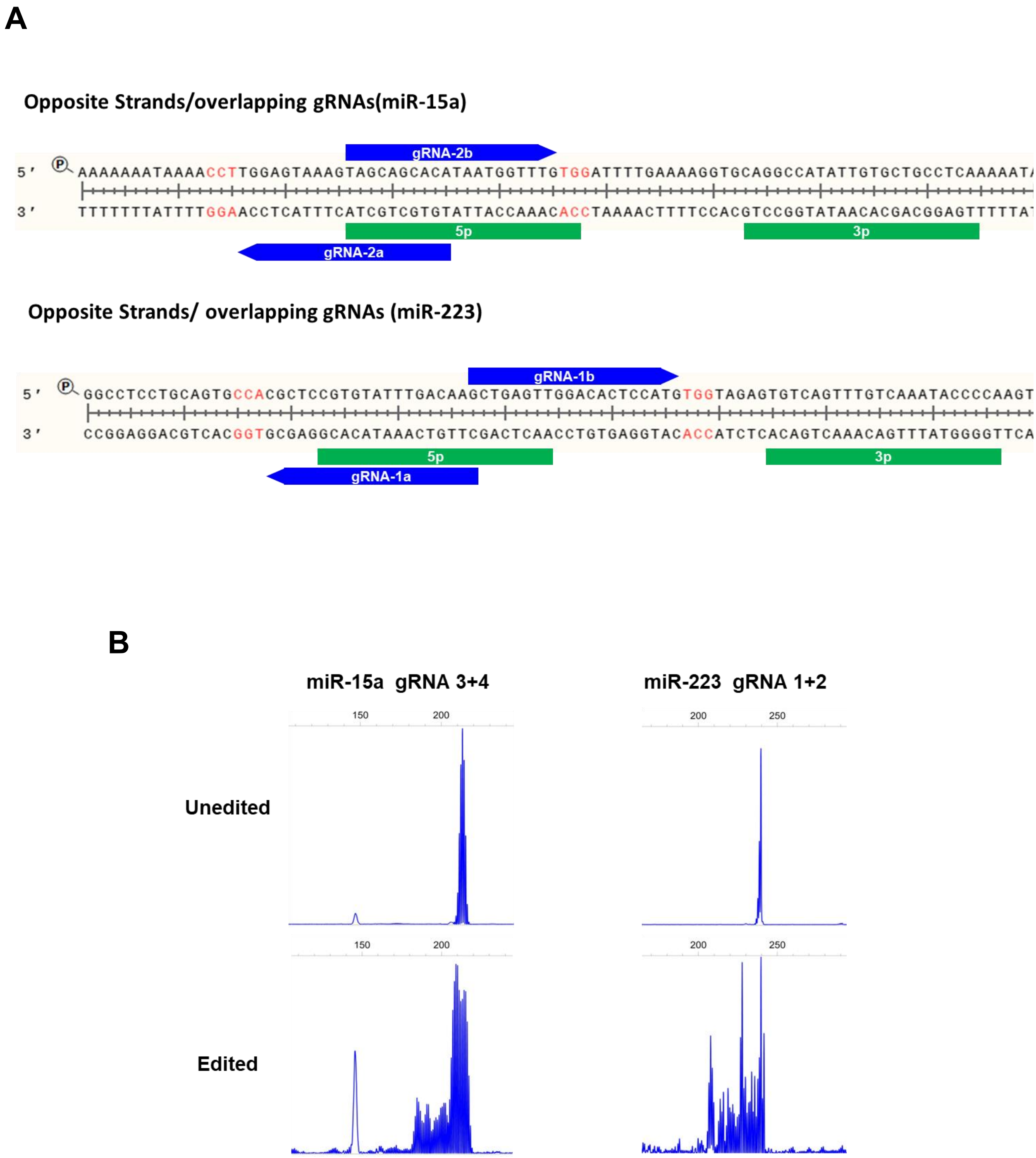

Supplementary Figure S7. (**A**) Two pairs of overlapping dgRNAs with low gRNA offsets and (**B**) Results of FL-PCR-CE analysis indicating the formation of a substantial number of in-dels without significant deletion formation between the gRNAs.

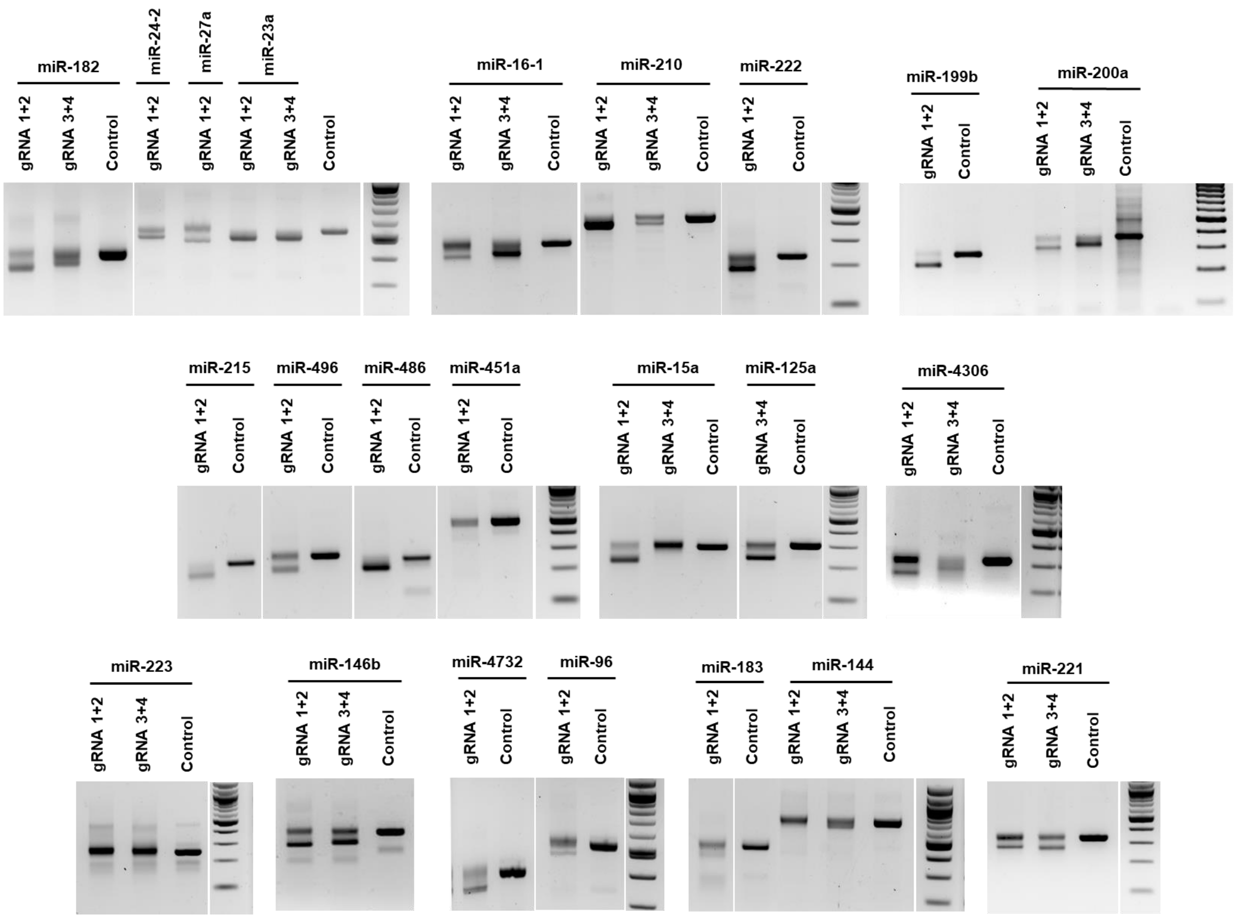

Supplementary Figure S8. Agarose gel electrophoresis (AGE) analysis of the PCR-amplified products obtained using primers specific to the regions targeted by dgRNAs for miRNAs. The miRNAs were targeted with a single pair of gRNAs (gRNA1+ gRNA2) or with two pairs of gRNAs (gRNA1+ gRNA2 and gRNA3+ gRNA4).

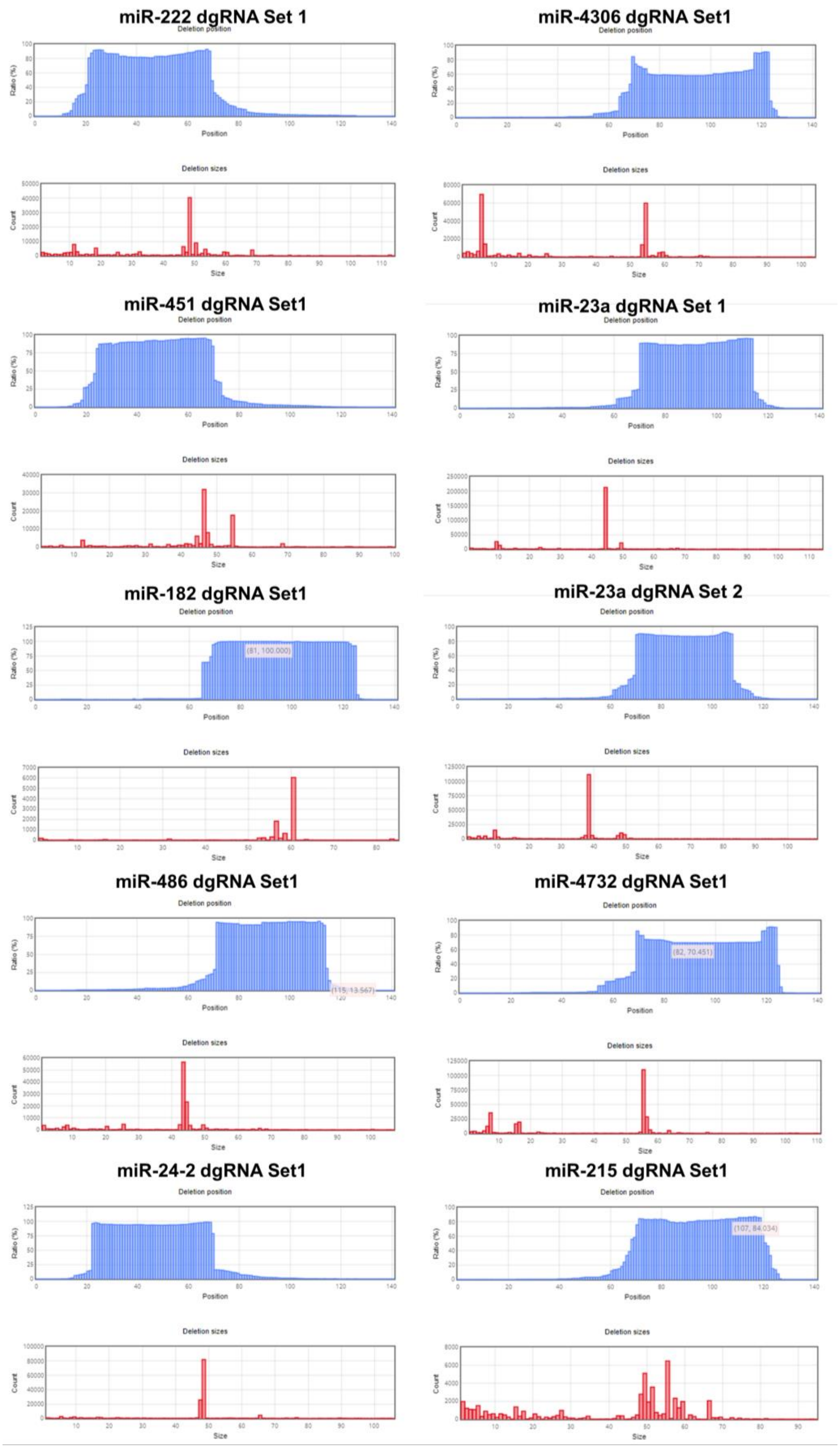

Supplementary Figure S9. NGS analysis of the PCR amplified products showing efficient formation of deletions at the dgRNA targeted miRNA regions.

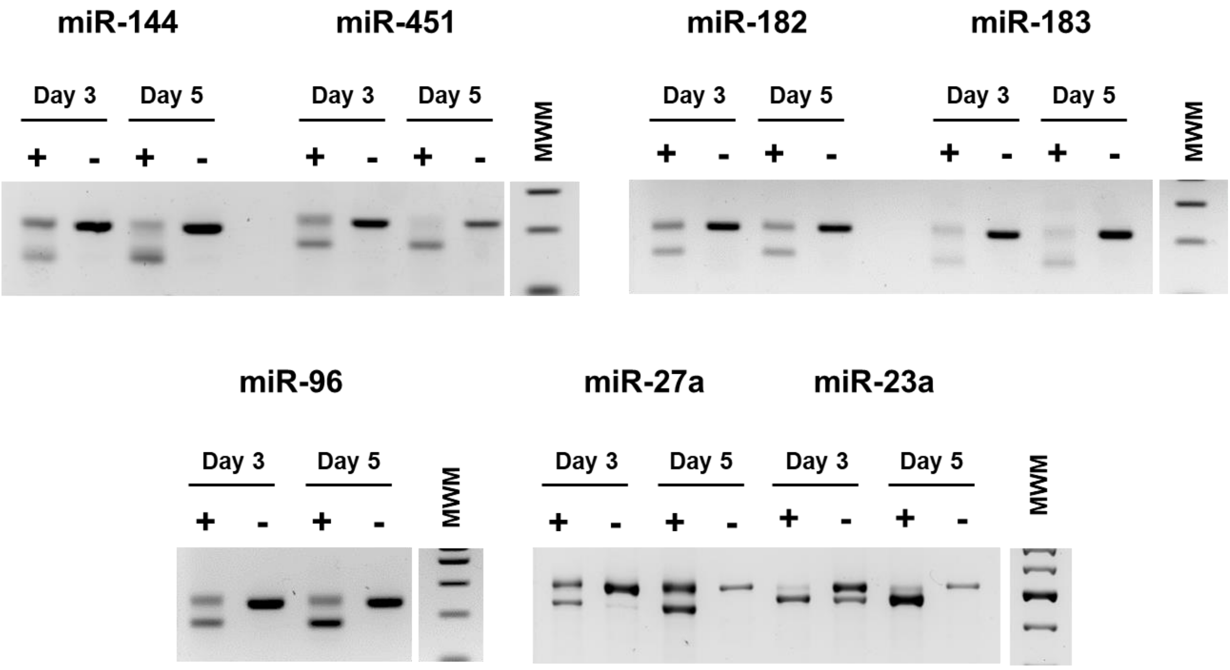

Supplementary Figure S10. Agarose gel electrophoresis (AGE) of PCR-amplified products from dgRNA-targeted miRNA regions in iPSCs without Dox treatment and after Dox treatment for 3 and 5 days. MWM: Molecular weight marker.

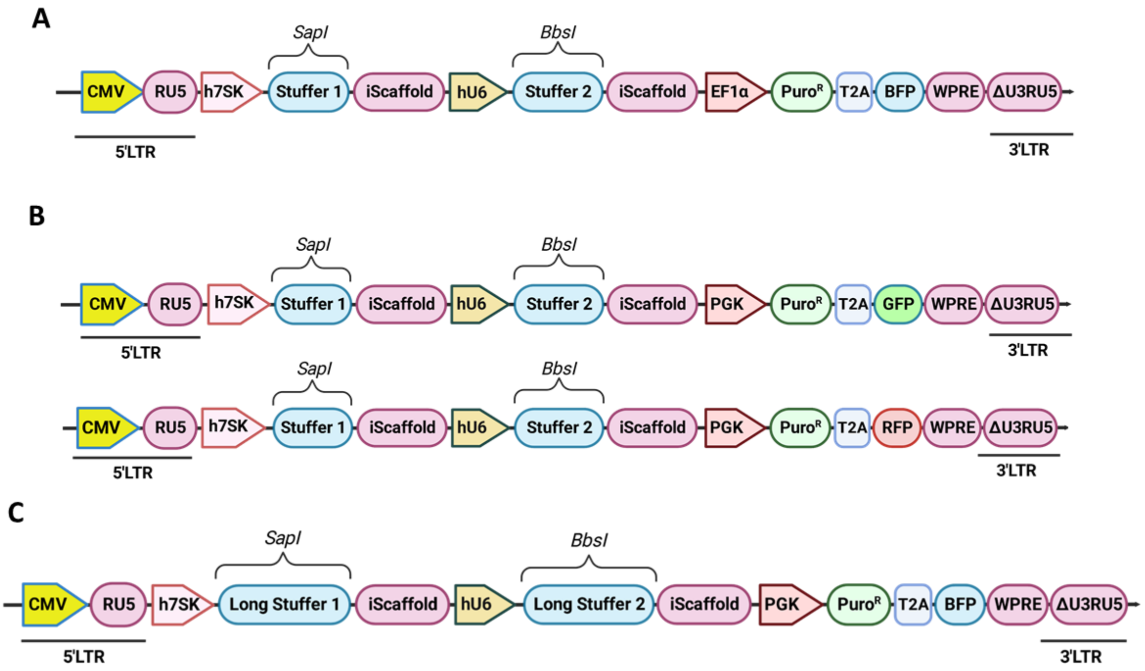

Supplementary Figure S11. Generation of different variants of pKLV2.2 dgRNA plasmids. pKLV2.2-h7SKgRNA5(SapI)-hU6gRNA5(BbsI)-PGKpuroBFP-W was modified to (A) replace PGK promoter with EF1a promoter to express puromycin resistance gene (PuroR) and BFP, (B) replace BFP with GFP and RFP selection markers and (C) replace short stuffer sequences with long stuffer sequences.

Supplementary Table S1. The number of sgRNAs targeting biogenesis sites and seed sequences of 30 miRNAs (The regions defined as biogenesis sites and seed sequences are depicted in Fig. 1A).

miRNA	5'DR	5' DC	3' DC	3' DR	5p miRNA seed	3p miRNA seed
miR -141	-	-	-	1	1	-
miR-182	1	1	-	-	-	-
miR-183	1	-	-	-	-	-
miR-15a	-	-	-	-	-	-
miR-16	-	-	-	-	1	-
miR-125a	-	1	-	-	1	1
miR-146b	-	-	-	1	-	3
miR-199b	1	-	-	-	2	-
miR-200a	-	-	-	1	-	-
miR-210	-	-	-	-	-	2
miR-221	-	-	-	-	1	-
miR-222	-	-	-	-	-	-
miR-223	-	-	-	1	1	-
miR-144	-	-	-	-	-	-
miR-22	1	-	-	-	1	-
miR-4732	-	-	-	-	1	-
miR-486	-	1	1	-	1	1
miR-29b-1	-	-	-	1	-	-
miR192	-	-	1	-	-	-
miR-215	-	-	-	1	-	-
miR-96	-	-	-	-	-	-
miR-624	-	1	1	-	-	-
miR-195	-	1	-	-	-	-
miR-625	-	-	-	-	-	-
miR-23a	-	-	-	-	1	-
miR-24-2	1	-	-	-	3	-
miR-27a	-	-	-	-	-	1
miR-548ad	-	-	-	-	-	-
miR-370	-	-	-	-	1	2
miR-155	-	-	-	-	-	1

Supplementary Table S2. Comparison of experimental and predicted on-target efficiencies of sgRNAs that targeted miRNA genomic regions or HBG1/HBG2 promoter regions. The sequences of gRNAs, their respective off-target and on-target efficiency scores and the experimentally determined in-del percentages (in-del%) are shown. Some miRNA genomic regions were analyzed by 2 sgRNAs. n.d.: in-del % could not be determined due to the sequence complexity of the PCR products, such as repeat sequences.

gRNA expression lentiviral vectors	Genomic Regions	gRNAs		Efficiency Scores		Experimental in-del %
				Off-Target (Hsu et al., 2013)	On-Target (Doench et al., 2016)	
pKLV2.2	miR-15a	gRNA1:	AGTAAAGTAGCAGCACATAA	82	45	n.d.
		gRNA2:	TAGCAGCACATAATGGTTTG	80	47	n.d.
	miR-16	gRNA1:	TTCAGCAGCACAGTTAATAC	91	44	100%
	miR-125a	gRNA1:	CTGAGACCCTTTAACCTGTG	86	69	100%
	miR-146b	gRNA1:	GCACCAGAACTGAGTCCACA	86	68	100%
		gRNA2:	GTGCTACAACATCAATGCCA	89	65	n.d.
	miR-199b	gRNA1:	GAACAGATAGTCTAAACACT	83	69	100%
	miR-200a	gRNA1:	TGTCTGGTAACGATGTTCAA	90	57	87%
		gRNA2:	AGGTGACCCGCCGCTCGCCG	98	60	100%
	miR-210	gRNA1:	CGGCAGTGCCTCCAGGCGCA	84	56	100%
		gRNA2:	ACCCGGACACGGGGCCAGGA	81	55	93%
	miR-221	gRNA1:	CAACAGCTACATTGTCTGCT	82	52	90%
	miR-222	gRNA1:	TCTGGCTACTGGGTCTCTGA	79	51	99%
	miR-223	gRNA1:	CTTGTCAAATACACGGAGCG	95	62	n.d.
LentiCRISPR V2	miR-141	gRNA1:	TCCATCTTCCAGTACAGTGT	82	55	95%
		gRNA2:	TAACACTGTCTGGTAAAGA	91	46	97%
	miR-182	gRNA1:	GGCAATGGTAGAACTCACAC	89	62	91%
		gRNA2:	GGTTCTAGACTTGCCAACTA	97	41	93%
	miR-183	gRNA1:	TCCTGTTCTGTGTATGGCAC	81	41	95%
		gRNA2:	CTCAGTCAGTGAATTACCGA	94	60	32%
pL-CRISPR.EFS.GFP	HBG1/HBG2 promoter	gRNA 1 :	TAGTCTTAGAGTATCCAGTG	44	88	97.0%
		gRNA 2:	AGAGTATCCAGTGAGGCCAG	25	64	92.5%
		gRNA 4:	CTTGACCAATAGCCTTGACA	46	90	55.0%
		gRNA 5:	CAAGGCTATTGGTCAAGGCA	44	87	90.0%
		gRNA 6:	CTTGTCAAGGCTATTGGTCA	46	92	87.1%
		gRNA 7:	GCTAAACTCCACCCATGGGT	46	92	70.0%
		gRNA 8:	TGGTCAAGTTTGCCTTGTCA	43	89	52.2%
		gRNA 9:	AGGCAAGGCTGGCCAACCCA	30	68	91.6%
		gRNA 10:	GCTATTGGTCAAGGCAAGGC	42	86	71.6%
		gRNA 11:	TTAGAGTATCCAGTGAGGCC	37	78	68.2%
		gRNA 12:	ACTGGATACTCTAAGACTAT	46	91	100.0%
		gRNA 13:	GTTTGCCTTGTCAAGGCTAT	46	91	100.0%

Supplementary Table S3: Details of gRNAs used for dgRNA targeting of 33 miRNA genomic regions, including their orientations relative to each other, PAM and gRNA offsets, relative strand positions they bound to, and deletion percentages determined by FL-PCR-CE. Some miRNA regions were targeted by two pairs of dgRNAs.

miRNA	gRNAs	PAM Offset	gRNA Offset	PAM Orientation	Strand	Deletion % (CE)
miR-144	Set 1	61	27	Out+Out	Opposite	49
	Set 2	62	28	Out+Out	Opposite	91
miR-182	Set 1	59	59	In+Out	Same	99.3
	Set 2	44	10	Out+Out	Opposite	100
miR-183	Set 1	69	35	Out+Out	Opposite	96
miR-4306	Set 1	54	20	Out+Out	Opposite	98.3
	Set 2	36	2	Out+Out	Opposite	96.5
miR-23a	Set 1	44	78	In+In	Opposite	100
	Set 2	38	38	Out+In	Same	100
miR-215	Set 1	50	50	In+Out	Same	97.5
miR-486	Set 1	45	11	Out+Out	Opposite	100
miR-496	Set 1	63	97	In+In	Opposite	96
miR-24	Set 1	48	14	Out+Out	Opposite	100
miR-27a	Set 1	67	101	In+In	Opposite	92
miR-451	Set 1	47	13	Out+Out	Opposite	95
miR-4732	Set 1	55	89	In +Out	Opposite	100
miR-96	Set 1	49	49	Out+In	Same	91
miR-15a	Set 1	54	54	Out+in	Same	97
	Set 2	30	Overlapping	Out+Out	Opposite	ND
miR-16-1	Set 1	62	28	Out+Out	Opposite	68
	Set 2	45	45	Out+in	Same	86
miR-125a	Set 1	63	63	Out+in	Same	95
miR-146b	Set 1	49	49	Out+in	Same	97.3
	Set 2	44	44	In+Out	Same	95
miR-199b	Set 1	46	12	Out+Out	Opposite	98.5
miR-200a	Set 1	65	31	Out+Out	Opposite	95
	Set 2	50	16	Out+Out	Opposite	98
miR-210a	Set 1	48	82	In+In	Opposite	95
	Set 2	53	53	Out+in	Same	70
miR-221	Set 1	64	64	In+Out	Same	95
	Set 2	71	71	In+Out	Same	98
miR-222	Set 1	48	14	Out+Out	Opposite	100
miR-223	Set 1	33	Overlapping	Out+Out	Opposite	ND

Supplementary Table S4. The genomic locus-specific forward and reverse primers used for the capillary electrophoresis and Sanger Sequencing.

Target	**Purpose**	**CE -forward primer (5' to 3')**	**CE-reverse primer (5' to 3')**
miR-15a	First round PCR	Tag-ATTCTTTAGGCGCGAATGTG	AGGCACTGCTGACATTGCTA
miR-16-1	First round PCR	Tag-CCATATTGTGCTGCCTCAAA	TGAAAAGACTATCAATAAAACTGAAAA
miR-125a	First round PCR	Tag-TCTGTCTCTGGCTCTCAGAATG	TGCTCTGGAGGAAGGGTATG
miR-146b	First round PCR	Tag-AACGGGAGACGATTCACAGA	GATGTGGGCCTGCAGAAG
miR-199b	First round PCR	Tag-CTCCAGGCTCCGGTTCTG	GGGGTTCTCGGATCTCCA
miR-200a	First round PCR	Tag-GTTCTTCCCTGGGCTTCC	CTGGACTTCGGAGCTGACAC
miR-210a	First round PCR	Tag-CTGAAGTTGGGCCGAGAG	CCTCCCCACGGTATCCAG
miR-221	First round PCR	Tag-TGATCCAAAGAAAATGTGCAA	GTGTGTGTGTGTGTGTGTGTGT
miR-222	First round PCR	Tag-TGATCCAAAGAAAATGTGCAA	CACCCTAGAACTTGACTCTCTCC
miR-223	First round PCR	Tag-CCCTCTAGGGTCACATCTCC	GTATCCAGCTGGCAGTCCAT
miR-183	First round PCR	Tag-AATGTGCTAGTGCCAAAATCG	GGGGGTAGAGACCGTAGCAG
miR-96	First round PCR	Tag-GTTTCAGACTCCCCCACCTC	TACCGAAGGGCCATAAACAG
miR-182	First round PCR	Tag-GGATGCAGGGAAACACAGAG	CCTGCAGGAAGGACCTTGT
miR-215	First round PCR	Tag-TCTTCTGTGTACCTGCCATTG	TTTTGTAACACCAAAAAGATCCA
miR-4306	First round PCR	Tag-AGGCCTGCATGAGGACTTTA	ACTGGCTGGTCATGAGAACA
miR-451	First round PCR	Tag-AAGCATTCTGGTTCTCATCTTT	CTCTGGAGCCTGACAAGGAG
miR-144	First round PCR	Tag-TTGGCCTCTGAAAGTGGTG	CCCTGGGTCCCTATGAGAT
miR-486	First round PCR	Tag-GTAACGGCCTGGTTAGTGCTG	ATCTCCAGCAGGTGTGTGTG
miR-496	First round PCR	Tag-TTGCCACCTTCCTGACTCTC	GGTGATCCACTTCCTTACCC
miR-4732	First round PCR	Tag-GCAGCATCTCTCCTGTCCTC	ATTACGCCATCTCTGGCTTG
miR-24-2	First round PCR	Tag-TCTGCTCCAAGCATCAGC	TCTCTTTCTCCCCTCCAGGT
miR-27	First round PCR	Tag-TCTGCTCCAAGCATCAGC	TCTCTTTCTCCCCTCCAGGT
miR-23a	First round PCR	Tag-TCTGCTCCAAGCATCAGC	TCTCTTTCTCCCCTCCAGGT
miR-23a	First round PCR	Tag-TCTGCTCCAAGCATCAGC	TCTCTTTCTCCCCTCCAGGT
FANCA	First round PCR	Tag-CGGACACCAGCTTCCTCTTA	CGAACCGACTTCTCTCCGTA
FANCB	First round PCR	Tag-GGGGACAAGGGGAGGAAATC	CTGGCGGGAGGTTTGGAG
FANCC	First round PCR	Tag-TGGCACATTCAGCATTAAACAT	TTGTTTCATAGAGACCACCCC
FANCD1	First round PCR	Tag-AACAGGAGAAGGGGTGACTG	CAGGCCAAAGACGGTACAAC
LEF1	First round PCR	Tag-ACACATTCTGATCCACCCAG	CTGGTTACTGCTGCTGTGTG
CTSG	First round PCR	Tag-TCAGGTTTGCTTCCCCAGAT	TCAGATCCAGAGTCCAGCAG
CBR1	First round PCR	Tag-CACGACCGCCAGACTCGA	CGTTGTTGACCAGCACGTC
miR-30b	First round PCR	Tag-GGTCTCACATTTCCAACAAACC	TTCCGATTGAGTCTTGCCTC
All Targets	Second round PCR	FAM-Tag seuence in the first round forward primer	Same reverse primers used for the first round PCR
All targets	Sanger sequencing of the first round PCR products	Tag seuence in the first round forward primer	Same reverse primers used for the first round PCR

Note: The target regions were amplified with the first round PCR primers and then subjected to second round PCR with a FAM labelled forward primer that binds to common tag sequence "cactctttccctacacgacgctcttccgatct" present in the first round forward primer. The same reverse primer used for the first round PCR was used for the second round.
